# Colorectal cancer cells secreting DKK4 transform fibroblasts to promote tumour metastasis

**DOI:** 10.1038/s41388-024-03008-1

**Published:** 2024-03-22

**Authors:** Xue Li, Yulin Chen, Ran Lu, Min Hu, Lei Gu, Qiaorong Huang, Wentong Meng, Hongyan Zhu, Chuanwen Fan, Zongguang Zhou, Xianming Mo

**Affiliations:** 1 Department of Gastrointestinal Surgery, Laboratory of Stem Cell Biology, State Key Laboratory of Biotherapy, West China Hospital, Sichuan University, Chengdu, 610041 China; 2 Department of Gastrointestinal, Bariatric and Metabolic Surgery, Research Center for Nutrition, Metabolism & Food Safety, West China-PUMC C.C. Chen Institute of Health, West China School of Public Health and West China Fourth Hospital, Sichuan University, Chengdu, 610041 China; 3 Institute of Digestive Surgery and Department of Gastrointestinal Surgery, West China Hospital, Sichuan University, Chengdu, 610041 China

**Keywords:** Prognostic markers, Gastrointestinal cancer

## Abstract

Wnt/β-catenin signalling is aberrantly activated in most colorectal cancer (CRC) and is one key driver involved in the initiation and progression of CRC. However, mutations of APC gene in CRC patients retain certain activity of APC protein with decreased β-catenin signalling and DKK4 expression significantly upregulates and represses Wnt/β-catenin signalling in human CRC tissues, suggesting that a precisely modulated activation of the Wnt/β-catenin pathway is essential for CRC formation and progression. The underlying reasons why a specifically reduced degree, not a fully activating degree, of β-catenin signalling in CRC are unclear. Here, we showed that a soluble extracellular inhibitor of Wnt/β-catenin signalling, DKK4, is an independent factor for poor outcomes in CRC patients. DKK4 secreted from CRC cells inactivates β-catenin in fibroblasts to induce the formation of stress fibre-containing fibroblasts and myofibroblasts in culture conditions and in mouse CRC xenograft tissues, resulting in restricted expansion in tumour masses at primary sites and enhanced CRC metastasis in mouse models. Reduced β-catenin activity by a chemical inhibitor MSAB promoted the CRC metastasis. Our findings demonstrate why reduced β-catenin activity is needed for CRC progression and provide a mechanism by which interactions between CRC cells and stromal cells affect disease promotion.

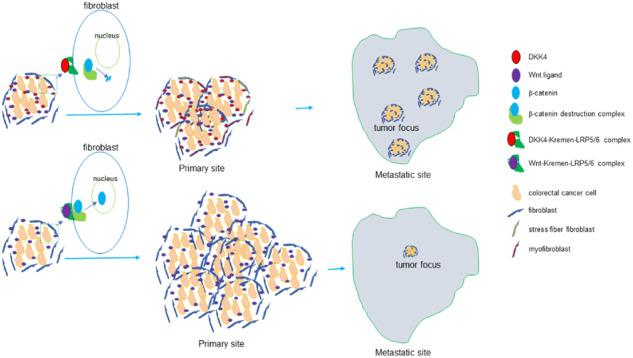

## Introduction

Dickkopf‐related protein 4 (DKK4) is a member of the Dickkopf family and is a soluble extracellular LRP5/6 antagonist that prevents the formation of the FRIZZLED (FZD)-LRP6 complex [1]. The FZD-LRP6 complex with Wnt ligands forms a ternary complex to promote the stabilization of β-catenin to activate the Wnt pathway [2]. The dynamic state of DKK4 itself and its interaction with LRP6 modulate Wnt pathways in cells. The DKK protein binds to Kremen to form a three-component complex with LRP5/6. The DKK-Kremen-LRP5/6 complex leads to rapid endocytosis and removal of LRP5/6 from the plasma membrane, resulting in inactivation of the Wnt/β-catenin pathway [3]. Human DKK4 is located on chromosome 8p11.2‐p11.1 and encodes a secreted DKK4 protein [4]. DKK4 is downregulated in hepatocellular carcinoma [5] and is upregulated in many other cancers, including ovarian cancer [6], renal cell carcinoma [7], gastric cancer [8], and colorectal cancer (CRC) [9]. DKK4 might be involved in the invasion of epithelial ovarian cancer [6], inhibits the invasion of hepatocellular carcinoma [10] and colorectal carcinoma, and suppresses colorectal cancer metastasis [11, 12]. DKK4 inhibition enhances chemosensitivity to current treatments in CRC [13] and non-small cell lung cancer [14]. Thus, DKK4 might act as a tumour suppressor gene or an oncogene in cancers.

CRC is one of the leading causes of cancer-related death worldwide. Mutational inactivation of the adenomatous polyposis coli (*APC*) tumour suppressor gene is known to be the initiating event in most sporadic and familial CRCs. Disruption of APC is linked to the activation of WNT pathways [15]. *WNT* was first identified to regulate *Drosophila* embryonic segment polarity and to be a proto-oncogene in breast tumours induced by mouse mammary tumour virus [16]. WNT has been implicated in diverse biological processes, including cell fate specification, cell proliferation, cell migration, dorsal axis formation, and asymmetric cell division [17]. The canonical Wnt signalling cascade is a multistep process that involves the relocalization, phosphorylation, and degradation of multiple proteins, culminating in a coordinated transcriptional response. Briefly, WNT ligands bind FZD and LRP receptor complexes, initiating membrane recruitment of key scaffold proteins and disruption of the β-catenin destruction complex (minimally composed of AXIN, APC, CK1, GSK3β) to release β-catenin. β-catenin accumulates in the cytoplasm and then translocates into the nucleus to drive the transcription of target genes by associating with transcription factors and coactivators [18]. The majority of CRCs have high levels of WNT pathway activity [19]. The accumulated evidence indicates that WNT activation is one key driver involved in the initiation and progression of CRCs [19]. Mutations of *AXIN* and β-catenin are found in only a small fraction (5–6%) of CRC cases. In contrast, up to 92% of sporadic CRCs contained at least one alteration in a known WNT regulator APC protein and showed a notable bias towards truncating mutations, such as retention of one or, less frequently, two of the same 20-amino-acid repeats in *APC* within a central 200 amino acid region of the protein, which downregulates β-catenin protein expression [20]. The selected mutations for APC genotypes that are likely to retain some activity with decreased β-catenin signalling [21–23]. The expression of DKK4, an inhibitor of β-catenin signalling, has been shown significantly to upregulate and to repress Wnt/β-catenin signalling in human CRC tissues. The observations suggest that a precisely modulated activation of the Wnt/β-catenin pathway is essential for CRC formation and progression. Thus, the underlying reasons why a specifically reduced degree, not a fully activating degree, of activation in Wnt/β-catenin signalling pathways, is needed in CRCs are unknown, which may impact our ability to exploit such alterations in the mechanisms by which CRC develops and progresses for therapeutic approaches for the diseases. Previously, we isolated CRC stem cells from patient samples and screened the gene expression in CRC stem cells [24–26]. The results showed that DKK4 highly expressed in CRC stem cells and greatly decreased its expression in cancer cells derived from the differentiation of CRC stem cells. The distinctive DKK4 expressing patterns in CRC cells drive us to address roles of DKK4, an inhibitor of Wnt/β-catenin signalling pathway, in CRC formation and progression. Our data provide evidence to show a mechanism by which reduced β-catenin activity is needed for the disease progression of colorectal cancers.

## Results

### DKK4 is an independent factor for poor outcomes in CRC patients

**Fig. 1:**
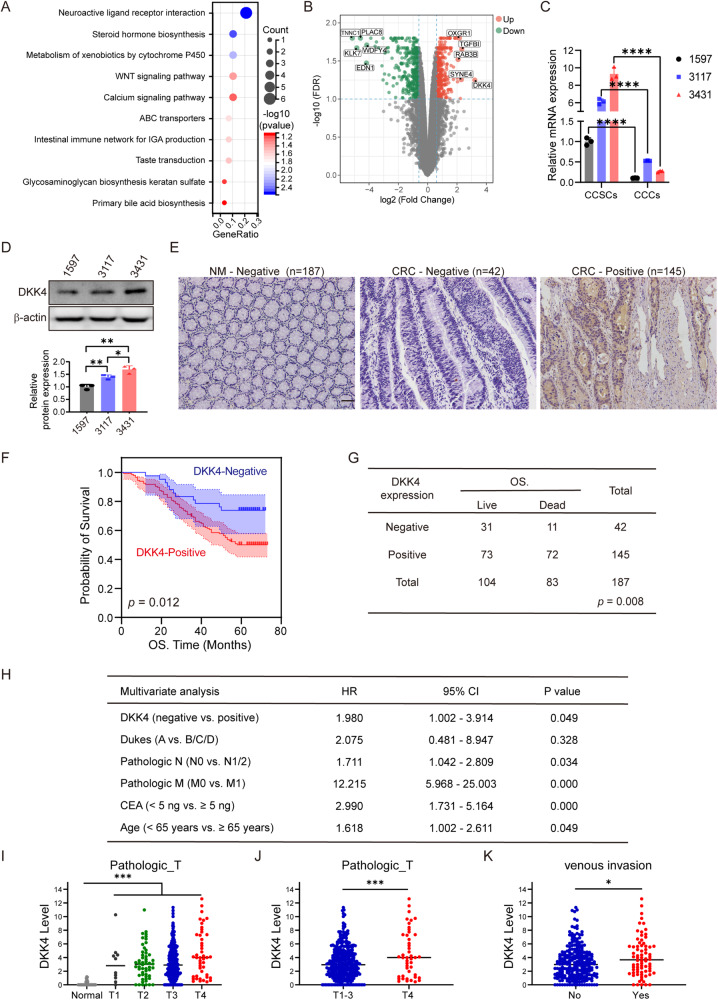
DKK4 is an independent factor for poor outcomes in CRC patients. **A** Top 10 pathways predicted to be most discrepant between CRC stem cells (CCSCs) and differentiated colorectal cancer cells (CCCs). **B** Volcano plot of gene expression in CCSCs versus CCCs. **C** DKK4 mRNA expression in each CCSCs and CCCs (*n* = 3). **D** Western blot images and quantification of DKK4 protein in each CCSCs (*n* = 3). **E** Representative immunohistochemical images of DKK4 expression in normal intestinal mucosa (NM) or CRC tissues (CRC) of 187 patients. Scale bar, 100 μm. **F, G** Kaplan–Meier curves (**F**) and univariate analysis (**G**) of DKK4 expression for overall survival in CRC patients from a Chinese cohort collected from 187 CRC patients who were diagnosed and operated on in West China Hospital between 2009 and 2010 (*n* = 187). **H** Multivariate analyses of DKK4 expression and clinical features for overall survival in CRC patients from a Chinese cohort (*n* = 187). **I,** **J** Correlation of DKK4 expression and pathological T stages in CRC tissue of patients from the TCGA database (dataset ID: TCGA.COADREAD.sampleMap/HiSeqV2, *n* = 358). **K** Correlation of DKK4 expression and venous invasion in CRC tissue of patients from the TCGA database (*n* = 358). *p < 0.05; **p < 0.01; ***p < 0.001; ****p < 0.0001 by Student’s unpaired t test unless stated otherwise.

After induction to differentiation of CRC stem cells from patients, the progenies of the colorectal stem cells were subjected to gene expression profiling. Many components of Wnt signalling pathways were substantially reduced in cancer cells derived from colorectal stem cells (Fig. 1A). Among them, DKK4 expression was decreased mainly in differentiated cancer cells (Fig. 1B, C). RT‒PCR and Western blotting results showed that DKK4 was expressed in CRC stem cells from the samples of three patients and the CRC cell lines SW480, SW620, and HCT116 (Fig. 1C, D; and Supplementary Fig. S1A‒G). To identify potential correlations with the outcomes of CRC patients, we analyzed DKK4 protein expression in sections of CRC tissues and matched colorectal mucosa tissues resected from 187 CRC patients. Immunohistochemical staining revealed that DKK4 expression was negative in the colorectal mucosa and positive in the CRC tissues of 145 patients (Fig. 1E). Next, we investigated whether DKK4 expression was associated with the prognosis and/or clinical characteristics of 187 CRC patients. Kaplan‒Meier analysis showed that DKK4 expression was correlated with poor overall survival (Fig. 1F, Kaplan–Meier curves, *p* = 0.012; and Fig. 1G, univariate analysis, *p* = 0.008). Multivariate Cox regression analysis revealed DKK4 expression to be an independent prognostic factor for poor survival (Fig. 1H). The results are inconsistent with published data [12]. Thus, an analysis of mRNA expression data from The Cancer Genome Atlas (TCGA) (dataset ID: TCGA.COADREAD.sampleMap/HiSeqV2) were performed to verify our results and revealed that the level of DKK4 expression was higher in cancer tissues than in normal tissues and was positively correlated with advanced diseases (Fig. 1I; and Supplementary Fig. S1H‒J). Kaplan‒Meier estimation showed significantly shorter overall survival in colorectal adenocarcinoma patients with higher DKK4 gene expression (Supplementary Fig. S1H‒J). The expression level of DKK4 was also positively correlated with the pathological grade of CRC tissues and venous invasion of CRC (Fig. 1I‒K). An additional analysis of DKK4 mRNA expression data from another resource (dataset ID: TCGA.COAD.sampleMap/GAV2) revealed similar results. Thus, DKK4 is an independent factor for poor outcomes in CRC patients.

### DKK4 restricts the expansion of CRC xenografts in mice

**Fig. 2:**
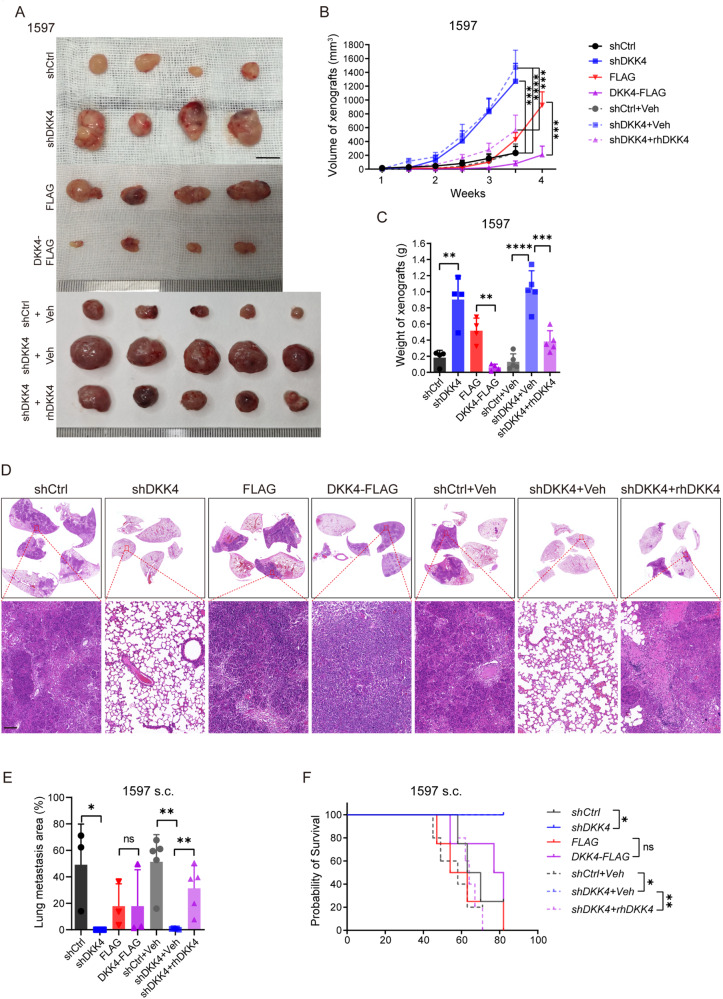
DKK4 restricts the expansion of CRC xenografts and promotes the metastasis of xenografts in mice. **A**–**C** Tumour images (**A**), tumour volume (mm3) change curves (**B**), and tumour weights (**C**) of harvested xenografts dissected from nude mice harbouring 1597-C-CSCs subcutaneously (s.c.) and treated with human recombinant DKK4 protein (20 mg/kg) or an equal volume of vehicle every 7 days (*n* ≥ 4). Scale bars, 10 mm. **D**–**F** H&E images and quantification of lung metastasis (**D**, **E**) and survival curves (**F**) of nude mice harbouring 1597-CCSCs s.c. and treated with human recombinant DKK4 protein (20 mg/kg) or an equal volumes of vehicle every 7 days (*n* ≥ 4). The dashed blue line of shDKK4+Veh overlapped with the solid blue line of shDKK4. Scale bars, 100 μm. Mean ± SD, *p < 0.05; **p < 0.01; ***p < 0.001; ****p < 0.0001 by Student’s unpaired t test (**B**, **C**), Mann‒Whitney *u* test (**E**) and log-rank test (**F**).

To address the roles of DKK4 in CRC progression, we employed shRNA to knock down DKK4 expression in CRC stem cells derived from samples of three patients and cancer cell lines, including SW480 and SW620 cell lines (Supplementary Fig. S1A‒C; and Supplementary Fig. S1E‒G). Moreover, cancer stem cells from the three patients and the HCT116 cancer cell line were infected with lentiviruses carrying the DKK4 coding sequence for overexpression and the corresponding control lentiviruses (Supplementary Fig. S1D‒G). After the evaluation of DKK4 expression (Supplementary Fig. S1A‒G), cancer stem cells and cancer cell lines with reduced expression or overexpression of DKK4 were subcutaneously implanted in the flank region of athymic nude mice. The mice with cancer stem cells from the samples of two patients (1597, 3431) showed a large increase in tumour volume as well as tumour weight when DKK4 expression was knocked down in the cancer cells compared to those of the shRNA control groups (Fig. 2A‒C; and Supplementary Fig. S2A‒C). The cancer stem cells from the samples of one patient (3117) did not result in obvious alterations in growth in mice after DKK4 expression was reduced by knockdown (Supplementary Fig. S2D‒F). The cancer cell lines SW480 and SW620, with reduced DKK4 expression, were subcutaneously injected into mice. A large increase in tumour volumes as well as tumour weights of xenografts was observed in the mice injected with SW620 cells carrying DKK4 shRNA (Supplementary Fig. S2G‒I). In contrast, reduced expression of DKK4 by knockdown did not increase the masses of xenografts in the mice injected with SW480 cells (Supplementary Fig. S2J‒L). The cancer stem cells and cancer cell line HCT116 with DKK4 overexpression substantially reduced the tumour volume and tumour weight in mice (Supplementary Fig. S2M‒O). Administration of the human DKK4 recombinant protein into the xenograft sites restored the effects of reduced DKK4 expression by knocking down tumour growth of cancer stem cells from the patient sample (1597) in mice (Fig. 2A‒C). Thus, the reduced expression of DKK4 promotes the expansion of xenografts in two CRC stem cells tested (1597 and 3431) and one CRC cell line (SW620). The increased expression of DKK4 restricts the expansion of xenografts derived from all cancer cells tested. The results indicate that differential effects of DKK4 on the growth of primary CRC cancers might be that different types of CRC cancers are respectively required a specific activation of Wnt/β-catenin signalling pathways. As mentioned above, detectable DKK4 protein levels are correlated with a negative effect on the overall survival of CRC patients. The phenotypes of CRC xenograft experiments in mice are inconsistent with the outcome of CRC patients.

### A proper level of DKK4 promotes the metastasis of CRC xenografts in mice

Metastasis has been shown to be a major factor for poor outcomes in CRC patients. Thus, we tested whether DKK4 plays roles in CRC metastatic processes. First, cancer stem cells (1597) and cancer cell lines (SW480) with reduced expression or overexpression of DKK4 were intravenously administered to mice. The results showed that DKK4 did not show any alteration in metastatic abilities in mice (Supplementary Fig. S3A‒D). The intrasplenic injection with two cancer stem cells (3431 and 1597) showed similar results (Supplementary Fig. S3E, F). These results indicate that DKK4 expression does not directly alter the metastatic abilities of CRC cells and cancer stem cells.

For further determination of the metastatic abilities of cancer stem cells and cancer cell lines, cells with reduced DKK4 expression were intraperitoneally injected into mice. The examinations showed that the numbers and volumes of lung tumour foci were dramatically higher in the mice injected with 3117 cells with control shRNA than in the mice injected with cells with shDKK4 (Supplementary Fig. S3G, H). The survival time of the control group was much shorter than that of the shDKK4 group (Supplementary Fig. S3I). The phenotypes in which reduced DKK4 expression attenuated lung metastasis were also demonstrated by tests of 3431 and SW620 cells (Supplementary Fig. S3J, K).

In previous experiments, subcutaneous CRC xenografts quickly reached large masses. Due to ethical principles, the mice were sacrificed before lung metastatic masses were detected. Therefore, we subcutaneously injected decreased cell numbers into nude mice. The results showed that the numbers and volumes of tumour masses in the lungs were dramatically higher in the mice injected with cancer cells (1597, 3117 and 3431) with control shRNA than in the mice injected with cells with reduced DKK4 expression (Fig. 2D, E; and Supplementary Fig. S3L, M). The survival time of the control group was much shorter than that of the shDKK4 group (Fig. 2F). Administration of the human DKK4 recombinant protein into the xenograft sites diminished the effects of shDKK4 on lung metastasis and the survival time of xenografts generated from 1597 cells in mice (Fig. 2D‒F). Reduced expression of DKK4 was able to inhibit CRC metastasis by subcutaneous inoculation and intraperitoneal inoculation but not by intravenous or intrasplenic injection, suggesting that the function of DKK4 has no significant effects on the migration, the survival in the blood circulation, and distal colonization of CRC cells and might mainly display roles in the primary tumour tissues to promote CRC metastasis.

Cancer stem cells and cancer cell lines overexpressing DKK4 were intraperitoneally and subcutaneously injected into mice. The results showed that the lung metastases and the survival time of the mice injected with DKK4-overexpressing cells were not obviously altered compared to those of the mice injected with control vectors (Fig. 2D‒F; and Supplementary Fig. S3G‒I). All the results indicate that the DKK4 level plays an essential role in the metastatic abilities of CRC xenografts in mice. The fact that a proper degree of DKK4 expression increases the metastatic abilities of CRC cells is consistent with the clinical outcomes of CRC patients.

### DKK4 secreted from CRC cells transforms fibroblasts in stromal tissues of xenografts in mice

To determine the underlying mechanism by which DKK4 promotes CRC metastatic abilities, we initially determined the characteristics of CRC stem cells and cancer cell lines in culture conditions. In vitro experiments showed that reduced DKK4 expression did not consistently alter the proliferation, apoptosis or migration of cancer stem cells and their differentiated progenies (Supplementary Fig. S4A‒J) or the ratios of cancer stem cells in xenografts (Supplementary Fig. S4K). These results are consistent with the observations that DKK4 does not directly modulate the metastatic abilities of CRC cells through intravenous and intrasplenic injections. All the results suggest that DKK4 displays essential functions in the microenvironments of cancer tissues for CRC metastatic abilities. The microenvironment of cancer includes blood vessels, neural tissue, stromal tissues, and immune cells [27]. Nude mice carry immunodeficiency of mature T cells and have few specific immune reactions [28]. We identified macrophages instead of all immune cells in the xenografts and found that macrophages were not altered in the CRC xenografts generated with CRC stem cells and cancer cell lines with reduced expression of DKK4 (data not shown). Then, we examined endothelial cells and neurons, the cellular components of blood vessels and neural tissues, in CRC xenografts. The results showed that there was no difference among the xenografts generated from CRC stem cells and cancer cell lines with reduced expression of DKK4 (Supplementary Fig. S5A‒F). All the tests indicate that the expression of DKK4 in CRC cells could not change the distributions and features of blood vessels, neural tissues, and immune cells in the microenvironments of cancer tissues.

**Fig. 3:**
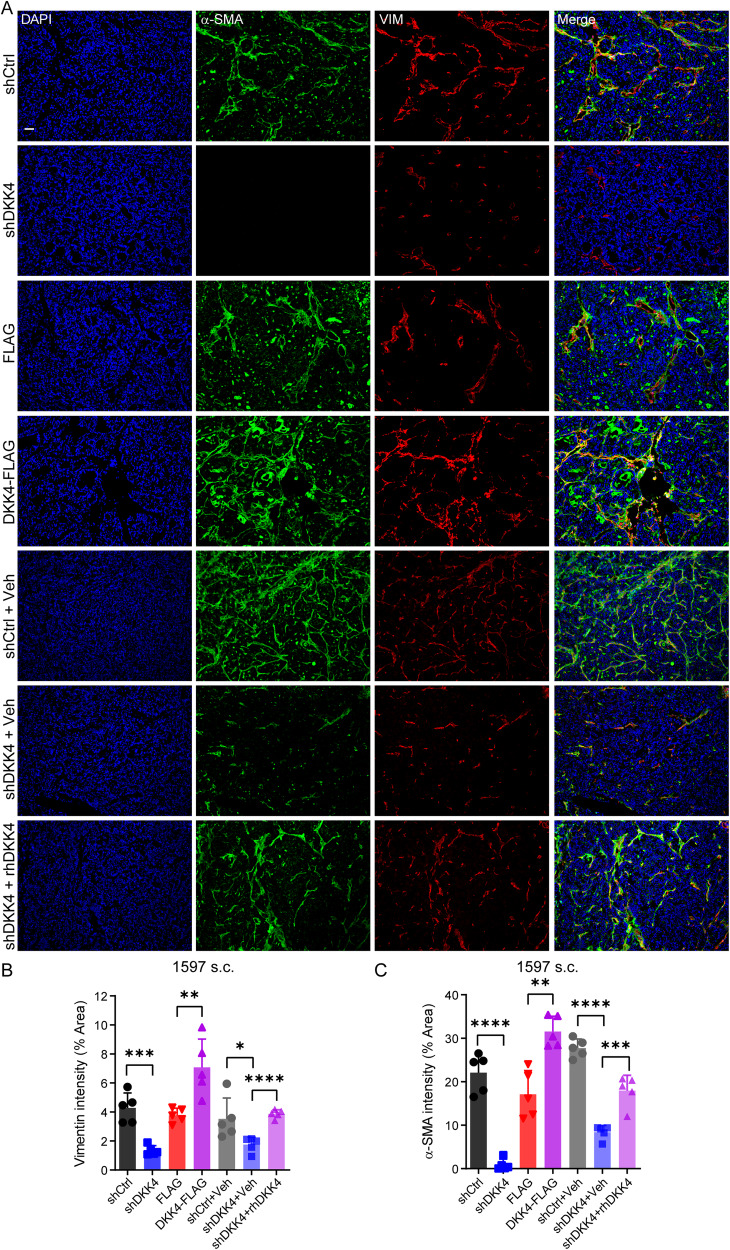
DKK4 secreted from CRC cells transforms fibroblasts in stromal tissues of xenografts in mice. **A–C** Representative images (**A**) and quantification of Vimentin (red, **B**) and α-SMA (green, **C**) immunofluorescence staining in xenografts dissected from nude mice harbouring 1597-CCSCs s.c. and treated with human recombinant DKK4 protein (20 mg/kg) or an equal volume of vehicle every 7 days. Scale bars, 50 μm. At least 3 xenografts were performed in each group, and 3-5 images of each xenograft were taken randomly. Mean ± SD, *p < 0.05; **p < 0.01; ***p < 0.001; ****p < 0.0001 by Student’s unpaired *t* test.

The major cellular components of stromal tissues are fibroblasts [27]. Thus, the subcutaneous and intraperitoneal CRC xenografts were sectioned and stained with anti-Vimentin (a protein marker of fibroblasts) and α-SMA (a marker of myofibroblasts) [29] antibodies. The examinations showed substantially reduced Vimentin^+^α-SMA^+^ fibroblasts distributed in the stromal tissues of xenografts generated from CRC stem cells (1597) and cancer cell lines with reduced expression of DKK4 (Fig. 3A‒C), suggesting that reduced expression of DKK4 in colorectal cancer cells reduces myofibroblasts in tumour stromal tissues. To confirm these results, we assessed the class II nonmuscle myosin isoform protein MYH9, another marker of myofibroblasts [30], in xenograft sections. The results showed that CRC xenografts generated from cancer stem cells and cancer cell lines with reduced expression of DKK4 carried much fewer α-SMA^+^MYH9^+^ myofibroblasts in the tumour stromal tissues (Supplementary Fig. S5G‒I). Administration of recombinant human DKK4 (rhDKK4) protein restored the expression of α-SMA and Vimentin in xenografts generated from CRC stem cells with reduced expression of DKK4 by knockdown (Fig. 3A‒C). Overexpression of DKK4 in cancer stem cells and cancer cell lines greatly enhanced the number of Vimentin^+^α-SMA^+^ myofibroblasts distributed in the stromal tissues of CRC xenografts (Fig. 3A‒C). The results indicate that DKK4 expressed in CRC cancer cells strongly induces the formation of Vimentin^+^α-SMA^+^ fibroblasts and α-SMA^+^ MYH9^+^ myofibroblasts in cancer stromal tissues in mouse models.

### DKK4 secreted from CRC cells transforms fibroblasts in vitro

**Fig. 4:**
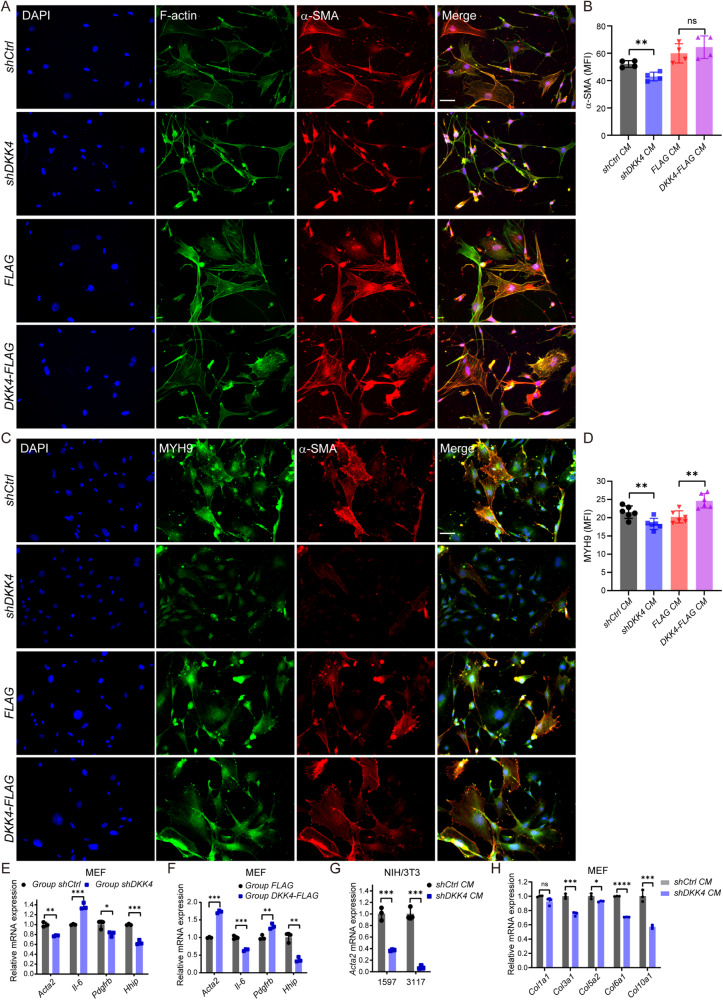
DKK4 secreted from CRC cells transforms fibroblasts in vitro. **A**, **B** α-SMA (red) immunofluorescence staining with F-actin (green) (**A**) and quantification (**B**) in MEFs cultured with conditioned medium (CM) obtained from 1597-CCSCs. At least three technical replicates were performed and 4-6 immunofluorescence images of each group were taken randomly. **C**, **D** MYH9 (green) immunofluorescence staining with α-SMA (red) (**C**) and quantification (**D**) in MEFs cultured with CM obtained from 1597-CCSCs. At least three technical replicates were performed and 4-6 immunofluorescence images of each group were taken randomly. **E**, **F** Myofibroblast-associated gene expression of MEFs cocultured with 1597-CCSCs. **E** shCtrl vs. shDKK4; **F** FLAG vs. DKK4-FLAG (*n* = 3). **G** *Acta2* expression in NIH/3T3 cells cultured with CM obtained from the respective CCSCs (*n* = 3). **H** Collagen expression of MEFs cultured with different CM obtained from the respective 1597-CCSCs (*n* = 3). Scale bars, 50 μm. MFI, mean fluorescence intensity. At least three independent experiments were performed for each assessment. Mean ± SD, *p < 0.05; **p < 0.01; ***p < 0.001; ****p < 0.0001 by Student’s unpaired t test.

Next, we obtained media from cancer stem cell and CRC cell line culture dishes and cultured mouse embryonic fibroblasts (MEFs), 3T3 fibroblasts, and human colon cancer-associated fibroblasts (CAFs) with conditioned culture media. The results showed that fibroblasts cultured in media from DKK4-expressing cancer stem cells and cancer cell lines presented a large, star shape and had cytoplasmic stress fibres. Actin, α-SMA, and MYH9 staining showed stress fibres in fibroblasts and myofibroblasts (Fig. 4A‒D; and Supplementary Fig. S6A‒F). In comparison, the fibroblasts cultured in the media from cancer stem cells and cancer cell lines with reduced expression of DKK4 had an elongated spindle shape with much fewer stress fibres and were small in diameter. The elongating fibroblasts were reduced with the expression of F-actin, α-SMA, and MYH9 proteins (Fig. 4A‒D; and Supplementary Fig. S6A‒F). The experiments of fibroblasts cocultured with CRC stem cells with or without the expression of DKK4 obtained the same results. Increased expression of DKK4 in cancer stem cells enhanced the expression of F-actin and MYH9 in fibroblasts but did not further change the morphologies of fibroblasts and myofibroblasts. The RNA expression results confirmed that reduced expression of DKK4 in cancer stem cells reduced α-SMA and collagen expression in fibroblasts (Fig. 4E‒H; and Supplementary Fig. S6G). Thus, DKK4 expression in cancer stem cells and CRC cell lines can transform fibroblasts into fibroblasts and myofibroblasts containing stress fibres.

### β-catenin modulates fibroblast transformation signalling via DKK4

**Fig. 5:**
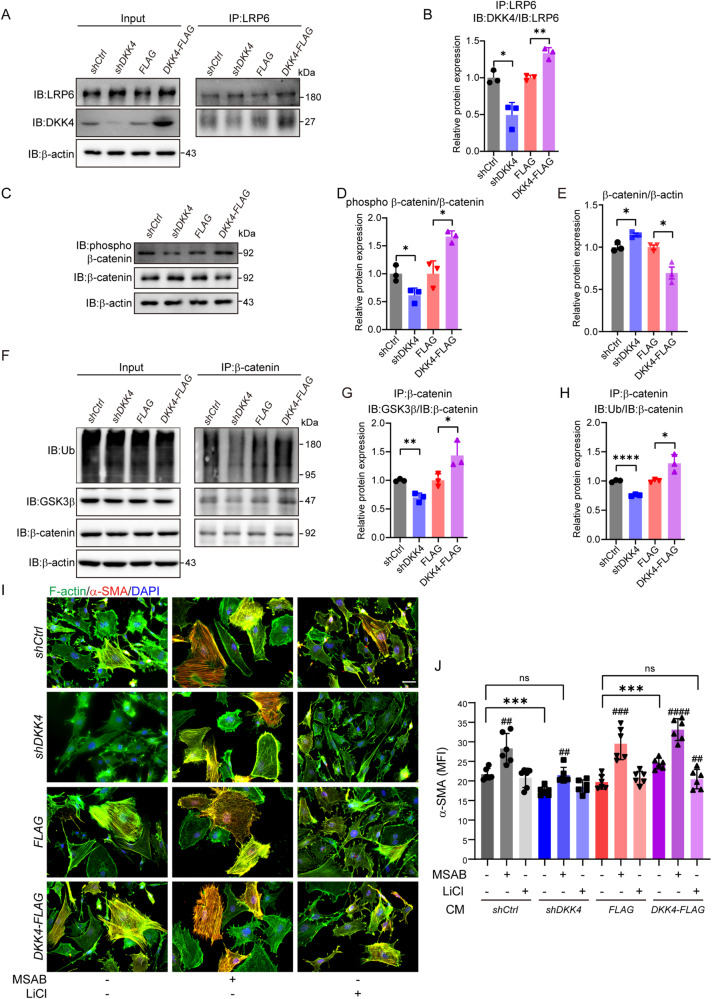
β-catenin modulates fibroblast transformation signalling via DKK4. **A**, **B** Western blot images (**A**) and quantification (**B**) of DKK4 by coimmunoprecipitation with LRP6 in MEFs cultured with CM obtained from the 1597-CCSCs. Statistical analysis was carried out with three technical replicates using each protein sample. **C**–**E** Western blot images (**C**) and quantification of the phosphorylation of β-catenin (Ser33/Ser37/Thr41) (**D**) and total β-catenin (**E**) in MEFs cultured with CM obtained from the 1597-CCSCs. Statistical analysis was carried out with three technical replicates using each protein sample. **F**–**H** Western blot images (**F**) and quantification of GSK3β (**G**) and ubiquitin (**H**) by coimmunoprecipitation with β-catenin in MEFs cultured with each CM obtained from the 1597-CCSCs and pretreated with 10 μM MG132 for 6 h. Statistical analysis was carried out with three technical replicates using each protein sample. **I**, **J** Representative images (**I**) and quantification (**J**) of F-actin (green) and α-SMA (red) double staining in MEFs cultured with (+) or without (-) WNT signalling modulators based on each CM obtained from 1597-CCSCs. MSAB, 2.5 μM; LiCl, 20 mM. Scale bars, 50 μm. MFI, mean fluorescence intensity. At least three independent experiments were performed. Mean ± SD, *p < 0.05; **p < 0.01; ***p < 0.001; ****p < 0.0001; ^##^*p* < 0.01; ^###^*p* < 0.001; ^####^*p* < 0.0001 by Student’s unpaired t test.

DKK4 is one component of the major Wnt/β-catenin signalling pathways [1, 10]. Thus, we first examined the gene activities involved in the Wnt/β-catenin signalling pathway in fibroblasts in culture conditions. The results showed that after downregulating DKK4 expression in the culture media of cancer stem cells and cancer cell lines, the expression of genes involved in the Wnt/β-catenin signalling pathway, including *Axin1*, *Axin2*, *Let*, and *Ctnnb1*, was highly enhanced in fibroblasts (Supplementary Fig. S7A‒C). Western blotting also showed that proteins involved in the Wnt/β-catenin signalling pathway, including Met and Cyclin D1, were increased in fibroblasts cocultured with cancer stem cells and cancer cell lines with reduced DKK4 expression (Supplementary Fig. S7D‒E). The total protein levels and the active form of β-catenin were significantly enhanced in fibroblasts cocultured with cancer stem cells and cancer cell lines with reduced expression of DKK4. Moreover, the total protein levels and active form of β-catenin were dramatically reduced in fibroblasts cultured in conditional media obtained from cultured cancer stem cells and cancer cell lines with overexpressing DKK4. Therefore, the mRNA expression or protein levels of genes involved in the Wnt/β-catenin signalling pathway in fibroblasts after culture with the overexpression of DKK4 in cancer stem cells and cancer cell lines were determined. DKKs were reported to bind to the WNT receptor LRP5/6 and inhibit Wnt signal transduction [31]. Coimmunoprecipitation experiments demonstrated that DKK4 bound to LRP6, which in turn led to the phosphorylation of β-catenin at Ser33/Ser37/Thr41 in fibroblasts (Fig. 5A‒E). Increased phospho-β-catenin was able to enhance the ubiquitination of β-catenin (Fig. 5F‒H). Increased ubiquitinated β-catenin promotes the degradation of β-catenin by the proteasome [31], resulting in reduced levels of β-catenin protein (Fig. 5C, E; and Supplementary Fig. S7D, E). The results suggest that the secretion of DKK4 from cancer stem cells and cancer cell lines can inhibit the Wnt/β-catenin signalling pathway via reduced β-catenin protein in fibroblasts.

To test whether the activity of β-catenin modulates the transformation of fibroblasts, we used a selective inhibitor of Wnt/β-catenin signalling, MSAB (methyl 3-{[(4-methylphenyl)sulfonyl] amino}benzoate) [32], to inhibit the activation of β-catenin in fibroblasts in culture. MSAB has been shown to bind to β-catenin, promoting its degradation, and specifically to downregulate Wnt/β-catenin target genes [32]. After treatment with MSAB, the fibroblasts showed a large star shape and had cytoplasmic stress fibres, and similar phenotypes were observed when the fibroblasts were cocultured with DKK4-expressing cancer stem cells and cancer cell lines (Supplementary Fig. S7F‒H). MSAB treatment was able to restore stress fibre expression in fibroblasts cultured with cancer cells carrying shDKK4 (Fig. 5I, J). Treatment with LiCl, a chemical activator of β-catenin [33], induced an elongated spindle shape and smaller fibroblasts with fewer stress fibres (Supplementary Fig. S7F‒H). LiCl treatment strongly reduced fibroblast transformation by DKK4-overexpressing cancer cells (Fig. 5I, J).

Then, we stained the sections of subcutaneous and intraperitoneal CRC xenografts with anti-β-catenin antibody. The examinations showed that β-catenin was highly enhanced in the stromal cells in CRC xenografts generated from cancer stem cells and cancer cell lines with reduced expression of DKK4 (Supplementary Fig. S7I, J). DKK4 overexpression in CRC cancer cells dramatically reduced the protein expression of β-catenin in stromal cells in CRC xenografts (Supplementary Fig. S7I, J). In contrast, the α-SMA^+^ fibroblasts were strongly negatively correlated with the expression of β-catenin in the stromal tissues in CRC xenografts (Supplementary Fig. S7I‒K). Together, these results indicate that DKK4-induced fibroblast transformation occurs via the inhibition of β-catenin signalling in fibroblasts.

### Α β-catenin inhibitor modulates fibroblast transformation and the metastasis of xenografts in mouse models

To address the activity of β-catenin in the transformation of fibroblasts in mice, we intraperitoneally injected MSAB into APC^min/+^ and nude mice. After 14 days, the skin and intestines were collected, sectioned, and stained with anti-Vimentin, F-actin, MYH9, and α-SMA antibodies. The Vimentin^+^ F-actin ^+^MYH9^+^ myofibroblasts were largely enhanced in the stromal tissues in the skin and intestines of the mice (Supplementary Fig. S8A‒H). However, the sizes and amounts of skin and intestinal stromal tissues were not altered by the MSAB treatments. Then, we intraperitoneally injected MSAB into nude mice with subcutaneous xenografts derived with CRC stem cells (3431). After 20 days of treatment with MSAB, the skin, intestines and xenografts were harvested, sectioned, and stained with Vimentin, F-actin, and α-SMA. The results showed that MSAB treatments enhanced Vimentin^+^ F-actin^+^ α-SMA ^+^ fibroblasts within the xenografts (Supplementary Fig. S9A‒D). The results confirm that the inhibition of β-catenin increases the transformation of fibroblasts in stromal tissues in tumour tissues and in mouse organs. Thus, the reduced activity of β-catenin can modulate the transformation of fibroblasts in vitro in culture conditions, in vivo in animal models and in tumour tissues.

**Fig. 6:**
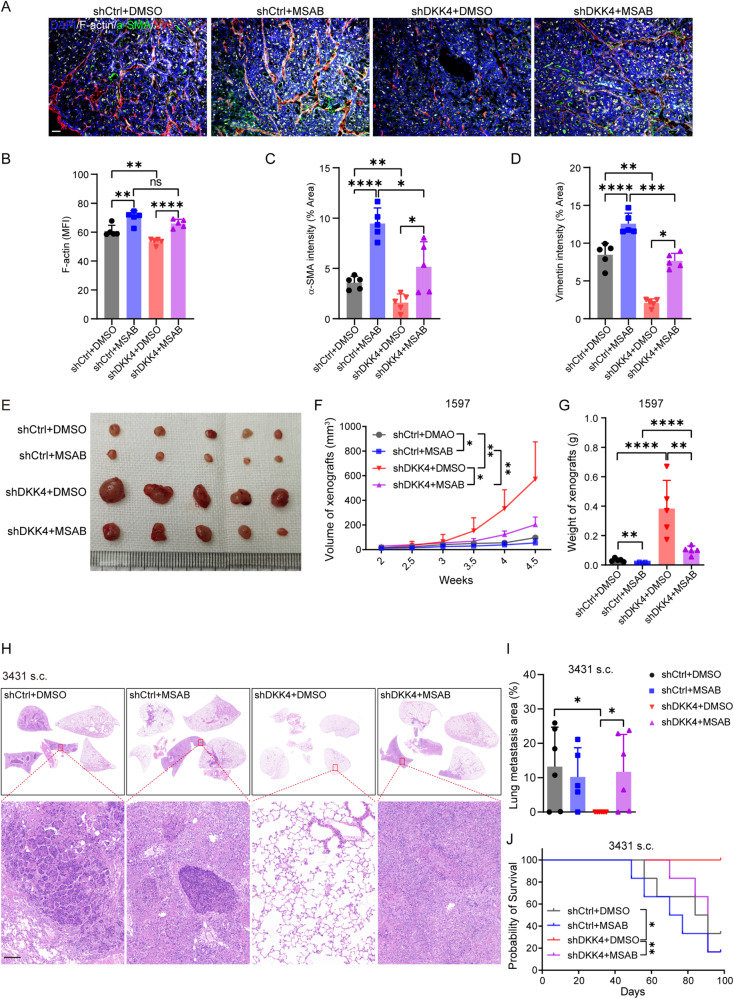
β-catenin inhibitor modulates fibroblast transformation and the metastasis of xenografts in mice models. **A**–**D** Immunofluorescence staining images (**A**) and quantification of F-actin (white, **B**), α-SMA (green, **C**) and Vimentin (red, **D**) triple staining in xenografts dissected from nude mice harbouring 1597-CCSCs s.c., and MSAB (20 mg/kg) or an equal volume of DMSO was administered every two days (n = 5). Scale bar, 50 μm. The multi-panel images were shown in Supplementary Fig. S9G. **E**–**G** Tumour images (**E**), tumour volume (mm3) changing curves (**F**), and tumour weights (**G**) of harvested xenografts dissected from nude mice harbouring 1597-CCSCs s.c. and administered MSAB (20 mg/kg) or an equal volume of DMSO every two days (*n* = 5). **H**–**J** H&E images and quantification of lung metastasis (**H**, **I**) and survival curves (**J**) of nude mice harbouring the respective 3431-CCSCs s.c. and administered MSAB (20 mg/kg) or an equal volume of DMSO every two days (*n* = 5). Scale bar, 100 μm. MFI, mean fluorescence intensity. Three independent experiments were performed for each assessment. Mean ± SD, *p < 0.05; **p < 0.01; ***p < 0.001; ****p < 0.0001 by Student’s unpaired *t* test (**B**, **C**, **D**, **F**, **G**), Mann‒Whitney *u* test (**I**) and log-rank test (**J**).

We wondered whether the transformation of fibroblasts by a β-catenin inhibitor in whole animal bodies affected the progression of CRC xenografts in mice. After intraperitoneal injection of MSAB into nude mice with subcutaneous xenografts implanted with CRC stem cells (3431), the xenografts were greatly reduced in growth, volume, and weight (Supplementary Fig. S9E, F). To evaluate whether MSAB treatment affected the lung metastatic capacity of CRC stem cells with reduced expression of DKK4, we subcutaneously injected decreased cell numbers of CRC stem cells (1597 and 3431) into nude mice. The results confirm that the inhibition of β-catenin increases the transformation of fibroblasts in stromal tissues and reduced the growth of xenografts (Fig. 6A‒G; and Supplementary Fig. S9G). MSAB treatment did not alter the lung metastatic capacity of CRC cells with normal DKK4 expression. In contrast, MSAB treatment greatly increased the lung metastatic capacity of CRC cells carrying shDKK4 and reduced the survival time of mice (Fig. 6H‒J; and Supplementary Fig. S9H, I). These results suggest that the transformation of fibroblasts by the reduced activity of β-catenin by an inhibitor in whole animal bodies can restrict the expansion of CRC tumours in mice and that the DKK4 change in cancer cells is able to alter the metastatic progression of CRC xenografts in mice.

### DKK4 antibodies modulate fibroblast transformation and the metastasis of xenografts in mouse models

**Fig. 7:**
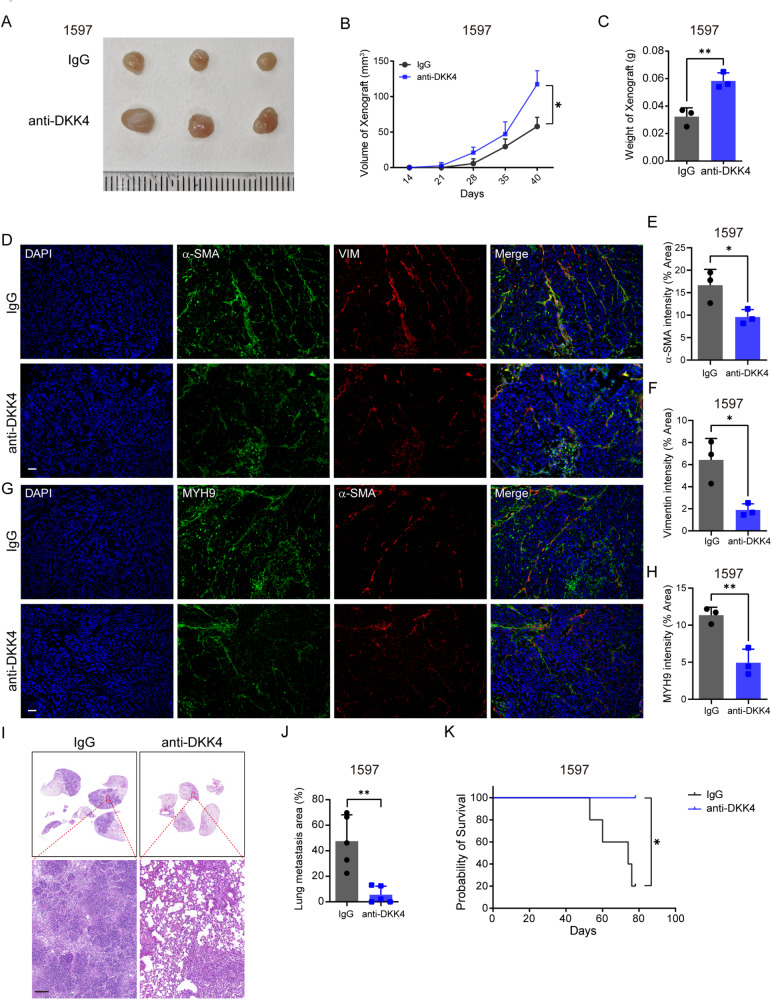
DKK4 antibodies modulate fibroblast transformation and the metastasis of xenografts in mouse models. **A**–**C** Tumour images (**A**), tumour volume (mm3) changing curves (**B**), and tumour weights (**C**) of harvested xenografts dissected from nude mice harbouring 1597-CCSCs subcutaneously and administered IgG isotype control or DKK4 antibody (100 mg/kg) every 7 days (*n* = 3). **D**–**F** Representative images (**D**) and quantification of α-SMA (green, **E**) and Vimentin (red, **F**) staining in xenografts of nude mice. Scale bar, 50 μm. **G**, **H** Representative images (**G**) and quantification of MYH9 (green, **H**) and α-SMA (red) staining in xenografts of nude mice. Scale bar, 50 μm. **I**–**K** H&E images and quantification of lung metastasis (**I**, **J**) and survival curves (**K**) of nude mice harbouring 1597-CCSCs subcutaneously and administered IgG isotype control or DKK4 antibody (100 mg/kg) every 7 days (*n* = 5). Scale bar, 100 μm. Mean ± SD, *p < 0.05; **p < 0.01 by Student’s unpaired t test (**B**, **C**, **E**, **F**, **H**, **J**), and log-rank test (**K**).

The therapeutic potential of DKK4 blockade in CRC metastasis was further evaluated. CRC stem cells (1597) were subcutaneously injected into nude mice, and DKK4 antibodies or IgG isotype control antibodies were subsequently administered into the xenograft sites every 7 days. The results showed that anti-DKK4 treatment promoted the growth of xenografts (Fig. 7A‒C) and reduced the distribution of Vimentin^+^ α-SMA^+^ MYH9^+^ myofibroblasts in the xenografts (Fig. 7D‒H). To determine whether DKK4 blockade could reduce the metastatic burden, we subcutaneously injected decreased numbers of 1597 cells into nude mice, and DKK4 antibodies or IgG isotype control antibodies were subsequently administered into the xenograft sites every 7 days for 11 weeks. The results showed that DKK4 blockade reduced the lung metastasis of CRC and increased the survival time of mice (Fig. 7I‒K). The results confirm that the transformation of fibroblasts by DKK4 at primary tumour sites restricts tumour growth and enhances the metastasis of CRCs. In addition, these data suggest that DKK4 blockade may be a potential therapeutic approach to block metastasis in CRC.

Thus, DKK4 expression in CRC cancer cells reduces the expression of β-catenin in stromal cells in cancer tissues, resulting in the transformation of fibroblasts to form stress fibre-containing fibroblasts and Vimentin^+^F-actin^+^MYH9^+^α-SMA^+^ myofibroblasts in cancer stromal tissues. The transformation of fibroblasts inhibits CRC expansion in local regions and promotes the remote metastasis of CRC.

## Discussion

Previous observation suggested that the survival time of CRC patients with high DKK4 expression was longer than that of patients with medium-low DKK4 expression [12]. However, we show here that DKK4 expression levels are negatively correlated with patient prognosis (Fig. 1F‒H; and Supplementary Fig. S1H‒J). Our results are contradicting to the previous observations [12] and indicate that DKK4 acts as a key factor of poor prognosis of patients with colorectal cancers and promotes the CRC metastasis in mouse models. In our studies, CCSCs (3117, 3431, and 1597) derived from samples of three patients were used to address the role of DKK4 in CRC. The results show that DKK4 expression significantly restricts the growth of xenografts generated by the CCSC 3431 and 1597 and does not display roles on the growth of xenografts generated by the CCSC 3117 in the primary regions. The lowest DKK4 protein level is detected in 1597. The DKK4 protein level of 3117 is lower than the one of 3431 (Fig. 1D). The differential growing phenotypes obtained from the three CCSCs in mice could not be explained by the DKK4 protein levels in CCSCs and may be due to the specific characteristics of the cancer tissues that are used to derive CCSCs. Indeed, 1597 was derived from the samples of colon adenocarcinoma with lower DKK4 expression (Supplementary Fig. S10A, B). 3431 was derived from a sample of rectal adenocarcinoma and 3117 was derived from a sample of rectal mucinous adenocarcinoma. The two tissue samples of rectal adenocarcinoma carried almost equal DKK4 protein levels (Supplementary Fig. S10A, B). The results suggest that DKK4 expression might inhibit the growth of some CRC tissues in the primary regions. Taken together, our results suggest that DKK4 acts as an oncogene in colorectal cancers. CRC cells secrete DKK4 to suppress the activity of β-catenin in fibroblast in stromal tissues. The inhibition of β-catenin function transforms fibroblasts and induces the differentiation of myofibroblasts. Transformed fibroblasts and myofibroblasts can constrain the expansion of tumour masses in local regions and promote remote metastasis of CRC cells. The signalling triggered by factors that mediate the communication between fibroblasts and myofibroblasts and CRC cells plays important roles during CRC initiation, progression, and therapeutic resistance [34]. Various Wnt ligands are secreted mostly by fibroblasts in cancer microenvironments [35, 36]. Fibroblast-derived hepatocyte growth factor (HGF) activates Wnt/β-catenin signalling and subsequently clonogenicity in cancer stem cells isolated from CRC patients and then promotes the development of CRC [37]. The Wnt ligand Wnt2 acts in an autocrine manner, generates morphogenetic changes in fibroblasts, and promotes the invasive and metastatic capacity of CRC-derived cells. The treatment of DLD1 and HCT116 cells, two CRC cell lines, with conditioned medium obtained from fibroblasts transfected with small interfering RNA targeting Wnt2 significantly reduced cell invasion and migration [35]. Aberrant accumulation of DKK4 promotes tumour progression via forming the immune suppressive microenvironment in gastrointestinal stromal tumour [38]. Meanwhile, DKK4 could inhibit osteoblast differentiation in MC3T3-E1 cells [39]. Taken together, our findings, combined with previous observations, indicate that the complex interactions between stromal cells and CRC cells lead to modulation of the activity of β-catenin in cancer cells and stromal cells and consequent effects on disease progression.

Our data show that fibroblasts and myofibroblasts containing stress fibres in cancer microenvironments can restrict the expansion of primary tumour masses and promote remote metastasis of CRC. Fibroblasts play key roles in cancer initiation and progression [40]. Elucidation of CAFs can offer prognostic and therapeutic value, especially in that a specific CAF population is associated with a particular cancer progression, which can help to provide proper therapies for specific cancer subtypes. However, CAFs have also been shown to have tumour-restraining functions. Various stromal transcriptional signatures in human cancer specimens and preinvasive or invasive malignancies have been associated with different stromal signatures [41]. Recently, the development of new culture models and the implementation of single-cell RNA-sequencing techniques have revealed CAF heterogeneities in many cancer types [42]. The discovery of CAF types may provide potential explanations for the controversy of CAFs playing both tumour-restraining and tumour-promoting functions in cancer. However, the roles of distinct CAF subtypes and how to selectively target these subtypes remain unclear. In this study, we show that DKK4 secreted from CRC cells enhances the formation of stress fibres containing fibroblasts. Many stress fibre-containing fibroblasts are Vimentin^+^F-actin^+^MYH9^+^α-SMA^+^ myofibroblasts. The transformation of fibroblasts and myofibroblasts in cancer tissues restrains the expansion of cancer masses in primary regions in mouse models and promotes the metastasis of CRC. However, further increasing transformation of fibroblasts and myofibroblasts by overexpressing DKK4 in cancer tissues could not enhance the metastasis of CRC (Fig. 2D, E; and Fig. 3A‒C). It may be a reason that enhancing stress fibre-containing fibroblasts and myofibroblasts had more restrictive effect on the growth of CRC tissues (Fig. 2A‒C; and Supplementary Fig. S2A‒F) and limit the cancer cells releasing from cancer tissues for remoting metastasis. The reduced number of CRC cells might decrease the enhanced remote metastasis by increasing formation of stress fibre-containing fibroblasts and myofibroblasts. Consequently, additionally increasing fibroblasts and myofibroblasts in cancer tissues could not enhance the metastasis. We also measured the production of extracellular matrix from DKK4-induced fibroblasts and myofibroblasts but did not detect significant alterations except for the production of a few collagens (Fig. 4H). After reviewing the literature, we realize that the results could not explain the mechanisms by which fibroblasts and myofibroblasts promote the metastasis of CRC. In the present work, we failed to determine how stress fibre-containing fibroblasts and myofibroblasts promote the metastasis of CRC. Next, new specific experiments, especially experiments to test the stress tension and contractile properties of fibroblasts and myofibroblasts, are needed to address these questions.

We show that the reduced activity of β-catenin modulates the transformation of fibroblasts into fibroblasts and myofibroblasts containing stress fibres in vitro and in animal models. Fibroblasts are widely distributed in stromal tissues and are needed for embryonic development, tissue homoeostasis, and tissue and organ repair and play critical roles in the initiation and progression of many diseases [43]. Fibroblasts are not terminally differentiated cells and have multidifferentiation potential depending on conditions in tissues and organs. A variety of signalling pathways promote the cellular differentiation of fibroblasts to produce myofibroblasts [43, 44]. Therefore, an understanding of the regulation of fibroblast differentiation is critical for promoting the functional repair of damaged tissues and limiting cancers and other diseases, including fibrosis. Regardless of the origin, increasing evidence strongly supports that the resultant myofibroblasts share the same properties and signalling cascade events that lead to their formation. The activity of Wnt/β-catenin signalling is implicated in the fibrogenesis of multiple organs [45, 46]. The increased nuclear accumulation of β-catenin was observed in human tissue samples from systemic scleroderma [47], idiopathic pulmonary fibrosis [48], and liver cirrhosis [49]. Wnt signalling stimulated by TGF-β1-induced inhibition of DKK1 has been shown to induce myofibroblast differentiation [50]. However, we tested fibroblasts cocultured with CRC stem cells and cancer cell lines and did not identify any altered activity of TGF-β1-induced signalling in fibroblasts. Thus, DKK4 secreted from CRC cells has no effect on TGF-β1 signalling when it transforms fibroblasts to myofibroblasts. Reduced β-catenin activity caused either by DKK4 or the β-catenin-inhibitor MSAB contributes to the differentiation of myofibroblasts. Next, how and under which conditions Wnt/ β-catenin signalling prevents the differentiation of fibroblasts to myofibroblasts should be intensively addressed.

## Materials and methods

### Animals

The animals were maintained and bred under specific-pathogen-free conditions, and all animal studies were carried out according to the animal protocol approved by the Sichuan University Institutional Animal Care and Use Committee. For tumour transplantation or drug treatments, only 5- to 6-week-old male nude mice were used.

### Patients and tissue samples

Expression of DKK4 protein and clinicopathological features of 187 primary CRC tissues and corresponding distant normal mucosa tissues were obtained from West China Hospital, Sichuan University. Informed consent was obtained from all patients. The protocol conformed to the Declaration of Helsinki, and all procedures were performed with the approval of the Institutional Review Board of West China Hospital, Sichuan University.

Moreover, the expression of DKK4 mRNA and clinicopathological features of TCGA-COREAD patients were obtained from the UCSC Xena Browser (https://xenabrowser.net/, dataset ID: TCGA.COADREAD.sampleMap/HiSeqV2), and 358 definite staging primary CRC tissues and 51 normal mucosal tissues were used for Kaplan‒Meier survival analysis.

### Cell culture and transduction

The CRC stem cells 1597, 3117 and 3431 were derived respectively from colon adenocarcinoma (1597), rectal adenocarcinoma (3431) and rectal mucinous adenocarcinoma (3117). The CCSCs were cultured in serum-free medium containing EGF (Peprotech, Cat# AF-100) and bFGF (Peprotech, Cat#100-18B) as described previously [24]. For in vitro differentiation, the culture medium was changed to high glucose DMEM (HyClone, Cat# SH30022.01B) with 10% foetal bovine serum (Gemini, Cat# 900-108).

The human intestinal cancer cell lines SW480, SW620 and HCT116 were acquired from ATCC and cultured according to the supplier’s instructions. The mouse fibroblast line NIH/3T3 was kindly provided by the Cell Bank/Stem Cell Bank, Chinese Academy of Sciences. Primary MEFs were obtained from 12- to 14-day-old foetuses of green fluorescent transgenic mice according to reported methods [51]. Primary human colon CAFs were obtained from fresh tumour tissue of a CRC patient according to reported methods [52]. Fibroblasts were cultured in high glucose DMEM (10% FBS). Third- or fourth-passage MEFs/CAFs were used for individual experiments.

Knockdown and overexpression of DKK4 were achieved by lentiviral infection. The sequence of DKK4 was obtained from the National Center for Biotechnology Information. The targeting sequences of each shRNA are listed in Supplementary Table S1. The plasmids and lentivirus for knockdown or overexpression of DKK4 were constructed as described elsewhere [53]. In brief, 293T cells were cotransfected with PSPAX2 and PMD2.G and respective lentiviral vectors. The supernatants were collected at 48 h and 72 h after incubation at 37°C and 5% CO_2_ saturation. Virus-containing supernatants of 293T cells were filtered through 0.45 μm filters and concentrated by PEG reagent (BioVision, Cat# K904-50). For lentivirus transduction, 1 × 10^6^ CRC cells were coincubated with lentivirus carrying shRNA constructs or the CDS of DKK4 overnight at 37°C. Then, the culture medium was replaced, and 5 μg/mL puromycin (InvivoGen, Cat# ant-pr-1) was used for positive transformant screening.

### Xenograft and metastasis model

Nude mice were randomly assigned to treatment groups and transplanted with CCSCs (1597, 3117 and 3431) and CRC cell lines (SW480, SW620 and HCT116). CRC cells (1‒3 × 10^5^) were suspended in PBS with or without Matrigel (Corning, Cat# 354262) and injected into 5-week-old nude mice subcutaneously, intraperitoneally, or intravenously (3-6 mice per group). Body weight and xenograft volume (if visible) were measured twice a week. MSAB (Selleck, Cat# S6901) administration was started at a dose of 10 mg/kg i.p. every 2 days. In situ subcutaneous administration of anti-DKK4 antibody (R&D Systems, Cat# MAB1269-500, 100 μg/kg) or human recombinant DKK4 protein (BIOINTRON, customized product, 20 μg/kg) was carried out every 7 days after CRC inoculation. When the established criteria for end-stage diseases were reached, mice were anaesthetized according to the 2020 AVMA Guidelines on Euthanasia state. The xenografts, lungs, livers, intestines and skins were dissected and fixed with 4% paraformaldehyde or Bouin’s fixative for subsequent analysis. Two experienced pathologists (blinded to the treatments of mice) assessed the metastasis of CRC independently. All animal experiments were approved by the Institutional Animal Care and Use Committee of Sichuan University. All staining was assessed by 3 persons with blinding.

### Conditioned medium experiments

Conditioned media of CRC cells were collected by centrifugation and immediately added to MEFs, CAFs or NIH/3T3 fibroblasts. For inhibition or activation of WNT signalling pathways, MEFs were treated with 2.5 μM MSAB (Selleck, Cat# S6901) or 20 mM LiCl (Sigma, Cat# L9650). Fibroblasts were allowed to grow for 3 days in conditioned medium before being harvested for further analysis.

### Cell proliferation and apoptosis analysis

Cells from different groups were dissociated with 0.25% trypsin-EDTA and harvested by centrifugation. Cell proliferation assays were performed using a Cell-Light EdU Apollo643 In Vitro Kit (RiboBio, Cat# C10310-2) following the manufacturer’s guidelines. Briefly, cells were incubated with 10 μM EdU for 2 hours and subsequently fixed in 4% paraformaldehyde. After Apollo 643 staining, cell nuclei were stained with Hoechst 33342, and cell proliferation was detected by a BD FACSCanto™ System (BD Biosciences, USA).

Cell apoptosis was determined using an Annexin V Apoptosis Detection Kit (KeyGEN, Cat# KGF004). Briefly, cells were incubated with 100 μL of binding buffer containing 2 μL of APC-conjugated Annexin V antibody and 1 μL of propidium iodide staining solution for 15 min at room temperature. After incubation, the cells were immediately analyzed by flow cytometry.

### Immunofluorescence and Imaging

Xenograft tissues for immunofluorescence were fixed with 4% paraformaldehyde for 1 h at room temperature, dehydrated with 30% sucrose and embedded with tissue-Tek OCT (Sakura, Cat# 4583). Then, OCT-embedded tissue samples were frozen and sectioned at 5 μm thickness immediately. Cells for immunofluorescence were fixed with 4% paraformaldehyde for 15 min at room temperature, washed with PBS and permeabilized with 0.5% Triton X-100 in PBS for 20 min. Thereafter, samples were blocked in PBS with 1% donkey serum (Solarbio, Cat# SL050) and 1% goat serum (Solarbio, Cat# SL038) for 1 h at room temperature. After blocking, the samples were incubated with primary antibodies specific for rabbit-anti-DKK4 (Abcepta, Cat# AP11649b, RRID: AB_10819953), rabbit-anti-α-SMA (Proteintech, Cat# 14395-1-AP, RRID: AB_2223009), mouse-anti-α-SMA (Proteintech, Cat# 67735-1-Ig, RRID: AB_2918504), rabbit-anti-Vimentin (Proteintech, Cat# 10366-1-AP, RRID: AB_2273020), mouse-anti-Vimentin (Proteintech, Cat# 60330-1-lg, RRID: AB_2881439), rabbit-anti-CD31 (Abcam, Cat# ab28364, RRID: AB_726362), chicken-anti-MAP2 (Aves Labs, Cat #: MAP, RRID: AB_2313549), mouse-anti-β-catenin (Santa Cruz, Cat# sc-7963, RRID: AB_626807), and mouse-anti-MYH9 (Proteintech, Cat# 60233-1-lg, RRID: AB_2881357), or Actin stain^TM^ 555 phalloidin (Cytoskeleton, Cat #PHDH1-A) overnight at 4 °C. Incubation of Alexa Fluor-conjugated secondary antibodies (Invitrogen) was carried out for 1 h at room temperature. DAPI was then used to counterstain the nuclei, and images were obtained by a Zeiss Axio Scope A1 ordinary polarizing microscope. At least 3 xenografts were stained in each group, and 3‒5 images of each xenograft were taken randomly. The images with large necrotic areas (a lot non-specific staining) were excluded for quantification. The quantification of the staining results was evaluated by positive area or mean fluorescence intensity (MFI) using ImageJ analysis software.

### Immunohistochemistry

Paraffin-embedded samples were sectioned at 5 μm thickness. Antigen retrieval was performed in a 95 °C water bath for 45‒60 min in 0.01 M citrate buffer (pH 6.0) to remove aldehyde links formed during the initial fixation of tissues. Tissue samples were blocked with 1% H_2_O_2_ for 20 min and incubated with antibodies specific for DKK4 (Abcepta, Cat# AP11649b, RRID: AB_10819953) overnight at 4 °C, and immunodetection was performed on the following day using DAB (Gene Tech, Cat# GK600510) according to the manufacturer’s instructions.

### Western blot

Protein was extracted from the cells using RIPA or IP buffer containing cocktails, resolved by SDS–polyacrylamide gels and then transferred to PVDF membranes. Primary antibodies against DKK4 (Abcepta, Cat# AP11649b, RRID: AB_10819953), β-catenin (Santa Cruz, Cat# sc-7963, RRID: AB_626807), nonphospho-β-catenin (Cell Signaling Technology, Cat# 4270, RRID: AB_1903918), phospho-β-catenin (Cell Signaling Technology, Cat# 9561, RRID: AB_331729), Met (Cell Signaling Technology, Cat# 8198, RRID: AB_10858224), Cyclin D1 (Cell Signaling Technology, Cat# 2978, RRID: AB_2259616), ubiquitin (Santa Cruz, Cat# sc-8017, RRID: AB_628423), LRP6 (Santa Cruz, Cat# sc-25317, RRID: AB_627894), and β-actin (Proteintech, Cat# 66009-1-lg, RRID: AB_2687938) were used. Peroxidase-conjugated secondary antibody (ZSGB-BIO, China) was used, and the antigen-antibody reaction was visualized by an automatic digital chemiluminescence imaging system (4600SF, Tanon, China). Band intensities were measured with ImageJ software. Statistical analysis was carried out with three technical replicates using each protein sample.

### Coimmunoprecipitation

For determination of whether DKK4 interacts with LRP6 and the changes in ubiquitination of β-catenin, MEFs were treated with different conditioned media for 48 hours and pretreated with 10 μM MG132 proteasome inhibitor (Selleck, Cat# S2619) for 6 h before being homogenized in IP buffer (50 mM Tris-HCl pH 7.4, 150 mM NaCl, 2 mM EDTA, 1% NP-40) containing protease inhibitor cocktail (Thermo Fisher, Cat# 78442). After centrifugation, the supernatant was collected and combined with beads (Beyotime, Cat# P2108) and antibodies against LRP6 (Santa Cruz, Cat# sc-25317, RRID: AB_627894) or β-catenin (Santa Cruz, Cat# sc-7963, RRID: AB_626807) overnight at 4 °C. After rinsing with wash buffer, subsequent analysis was performed according to the Western blotting method described above. Band intensities were measured with ImageJ software. Statistical analysis was carried out with three technical replicates using each protein sample.

### RNA purification and qRT-PCR

Cellular total RNA was extracted using TRIzol reagent (Molecular Research Center, Cat# TR118) according to the manufacturer’s instructions. Purified RNA was reverse transcribed using the Prime-Script RT Reagent Kit (TaKaRa, Cat# RR037A). Quantitative reverse transcription PCR (qRT-PCR) was performed using ChamQ SYBR qPCR Master Mix (Vazyme, Cat# Q311-02) according to the manufacturer’s instructions. The primer sequences are listed in Supplementary Table S2. Data were collected and analyzed with a CFX Connect Real-Time System (Bio-Rad, USA).

### Extreme limiting dilution analysis

Xenografts were collected and digested into single cells using collagenase type IV. Cells were stained with DAPI or 7AAD to exclude dead cells and seeded at a prescribed amount (1, 10, 100, 1000 cells/well) by a BD FACSAria II cell sorter. Cancer cells were cultured in 96-well or 24-well cell culture plates (BIOFIL, China) in serum-free medium as described above. After culture for 14 days, tumour spheres with diameters ≥ 75 μm were counted. More than six replicate wells were included in each analysis, and at least three independent experiments were conducted. Stem cell frequency was evaluated by Extreme Limiting Dilution Analysis software (ELDA, Walter and Eliza Hall Institute of Medical Research).

### Gene microarrays

mRNA microarray analysis was performed with 50 μg of total RNA using the Human 4 × 44 K Gene Expression Array (SHBIO, China). The data were analysed using an Agilent Microarray Scanner (Agilent technologies). Data were extracted with Feature Extraction software 10.7 (Agilent technologies). Raw data were normalized by Quantile algorithm, Gene Spring Software 11.0 (Agilent technologies). The microarray data were deposited in the public database. The differential expression of mRNAs was identified using the Limma package in R 3.6.0 and selected based on adjusted *p*-values (*p* < 0.1). The absolute value of log2 fold change was >1.5. Biological process, cell component, molecular function, and KEGG pathways were carried out using the clusterProfiler (version 3.14.3). The top 10 GO terms and pathways were visualized using the “ggplot2” package in R 3.6.0.

### Statistical analysis

All statistical analyses were performed with Prism 9.5 (GraphPad) and SPSS 22 (IBM). Estimating of variation within each group was performed before comparation. The variance similar between groups was statistically compared. Two-tailed unpaired Student’s *t* test, Mann‒Whitney *u* test, Chi-square test, and Kaplan‒Meier analysis were applied appropriately for statistical comparisons in this study. Data are presented as the mean ± SD or mean ± 95% confidence interval for a minimum of three independent experiments. Significant differences between the two groups are noted by asterisks (*p < 0.05; **p < 0.01; ***p < 0.001; ****p < 0.0001).

### Supplementary information

Supplemental figures and tables

## Data Availability

The microarray data has been deposited in the OMIX, China National Center for Bioinformation / Beijing Institute of Genomics, Chinese Academy of Sciences (https://ngdc.cncb.ac.cn/omix; accession no.OMIX005383). All data are available within the article, supplementary materials, or available from the corresponding author upon reasonable request.
