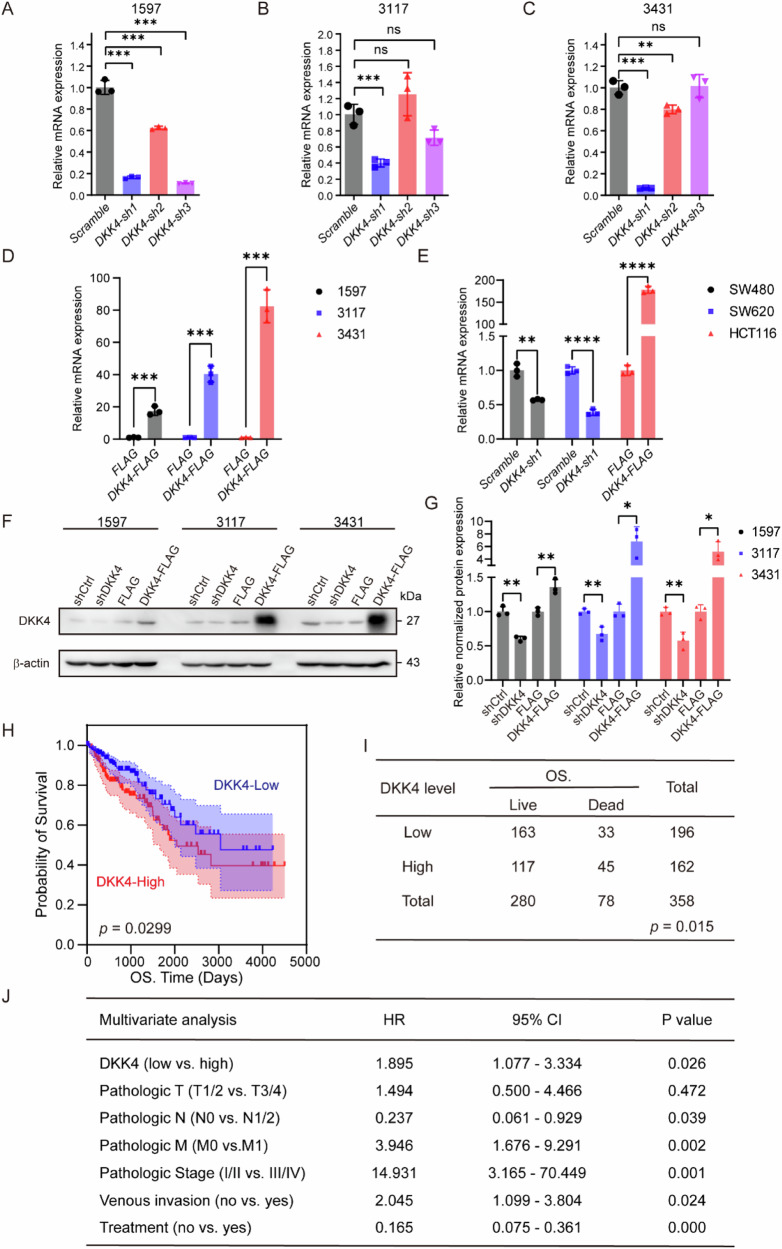# Correction: Colorectal cancer cells secreting DKK4 transform fibroblasts to promote tumour metastasis

**DOI:** 10.1038/s41388-024-03101-5

**Published:** 2024-07-15

**Authors:** Xue Li, Yulin Chen, Ran Lu, Min Hu, Lei Gu, Qiaorong Huang, Wentong Meng, Hongyan Zhu, Chuanwen Fan, Zongguang Zhou, Xianming Mo

**Affiliations:** 1 Department of Gastrointestinal Surgery, Laboratory of Stem Cell Biology, State Key Laboratory of Biotherapy, West China Hospital, Sichuan University, Chengdu, 610041 China; 2 Department of Gastrointestinal, Bariatric and Metabolic Surgery, Research Center for Nutrition, Metabolism & Food Safety, West China-PUMC C.C. Chen Institute of Health, West China School of Public Health and West China Fourth Hospital, Sichuan University, Chengdu, 610041 China; 3 Institute of Digestive Surgery and Department of Gastrointestinal Surgery, West China Hospital, Sichuan University, Chengdu, 610041 China

**Keywords:** Prognostic markers, Gastrointestinal cancer

Correction to: *Oncogene* 10.1038/s41388-024-03008-1, published online 22 March 2024

Following publication of this article, the authors noted an error in Supplementary Figure 1, whereby Fig. S1D was a copy of S1E. The correct Fig. S1D is provided below. The authors confirm this correction does not affect the results or conclusions of this work and wish to apologize for any inconvenience caused.

**Figure:**