# Supplementary material for: Colorectal cancer cells secreting DKK4 transform fibroblasts to promote tumour metastasis
Source: Oncogene. 2024 Mar 22;43(20):1506–21. doi: 10.1038/s41388-024-03008-1 (PMC11090838; doi:10.1038/s41388-024-03008-1)
Supplement: Supplementary file 1 — Supplemental figures and tables [file 41388_2024_3008_MOESM1_ESM.docx]

**Supplemental Material for**

**Colorectal cancer cells secreting DKK4 transform fibroblasts to promote tumour metastasis**

Xue Li^1, #^, Yulin Chen^1, #^, Ran Lu^1, #^, Min Hu^1^, Lei Gu^1^, Qiaorong Huang^1^, Wentong Meng^1^, Hongyan Zhu^1^, Chuanwen Fan^2, 3, *^, Zongguang Zhou^1, 3, *^, Xianming Mo^1, 4, *^

^1^Department of Gastrointestinal Surgery, Laboratory of Stem Cell Biology, State Key Laboratory of Biotherapy, West China Hospital, Sichuan University, Chengdu 610041, China.

^2^Department of Gastrointestinal, Bariatric and Metabolic Surgery, Research Center for Nutrition, Metabolism & Food Safety, West China-PUMC C.C. Chen Institute of Health, West China School of Public Health and West China Fourth Hospital, Sichuan University, Chengdu 610041, China.

^3^Institute of Digestive Surgery and Department of Gastrointestinal Surgery, West China Hospital, Sichuan University. Chengdu 610041, China.

^4^Leading contact

^#^These authors contributed equally to this work.

***Correspondence:**

Xianming Mo: xmingmo@scu.edu.cn

Zongguang Zhou: Zhou767@163.com

Chuanwen Fan: [chuanwen.fan@liu.se](mailto:chuanwen.fan@liu.se)

**This file includes:**

Figures. S1 to S10

Tables. S1 and S2

**Supplementary Figures**

**
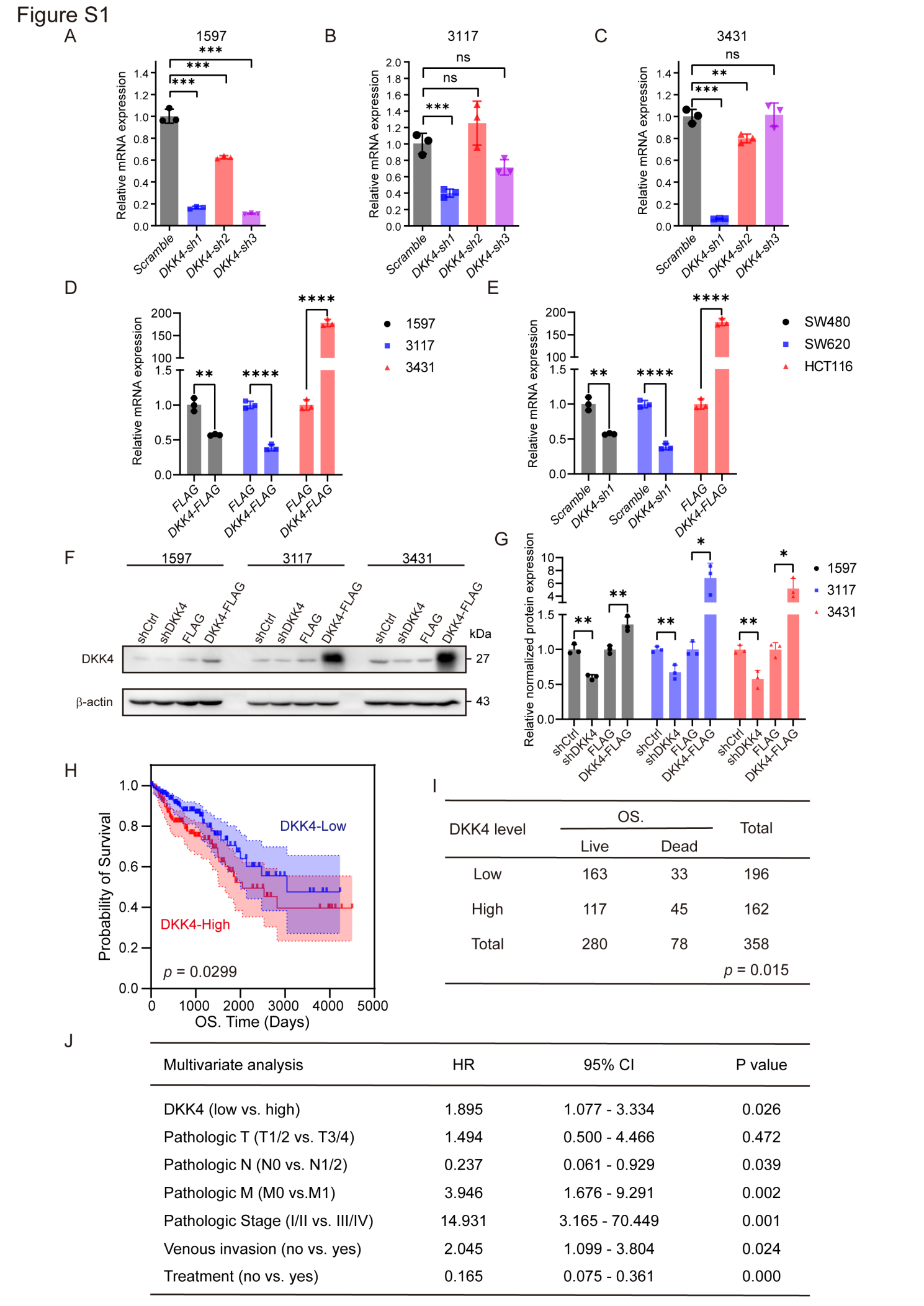
**

**Figure S1. DKK4 is an independent factor for poor outcome in CRC patients, related to Figure 1.**

(A-C) DKK4 mRNA expression in 1597 (A), 3117 (B), and 3431 (C) CCSCs with constitutive expression of different DKK4 shRNAs (scramble, sh1, sh2 or sh3) by lentivirus transduction (n = 3).

(D) DKK4 mRNA expression in each CCSCs constitutively expressing FLAG or DKK4-FLAG open reading frame by lentivirus transduction (n = 3).

(E) DKK4 mRNA expression in SW480, SW620 and HCT116 constitutively expressing different DKK4 shRNAs or open reading frame by lentivirus transduction (n = 3).

(F and G) Representative images (F) and quantification (G) of DKK4 protein expression from western blot performed by three CCSCs. Statistical analysis was carried out with three technical replicates using each protein sample.

(H and I) Kaplan–Meier curves (H) and univariate analysis (I) for overall survival of CRC patients from TCGA database (dataset ID: TCGA.COADREAD.sampleMap/HiSeqV2) according to DKK4 mRNA level. The optimal survival cut point was determined by X-Tile statistical software (n = 358).

(J) Multivariate analyses of DKK4 expression and clinical features for overall survival in CRC patients from TCGA database (n = 358).

Mean ± SD, *p < 0.05; **p < 0.01; ***p < 0.001 by Student’s unpaired t test unless stated otherwise.


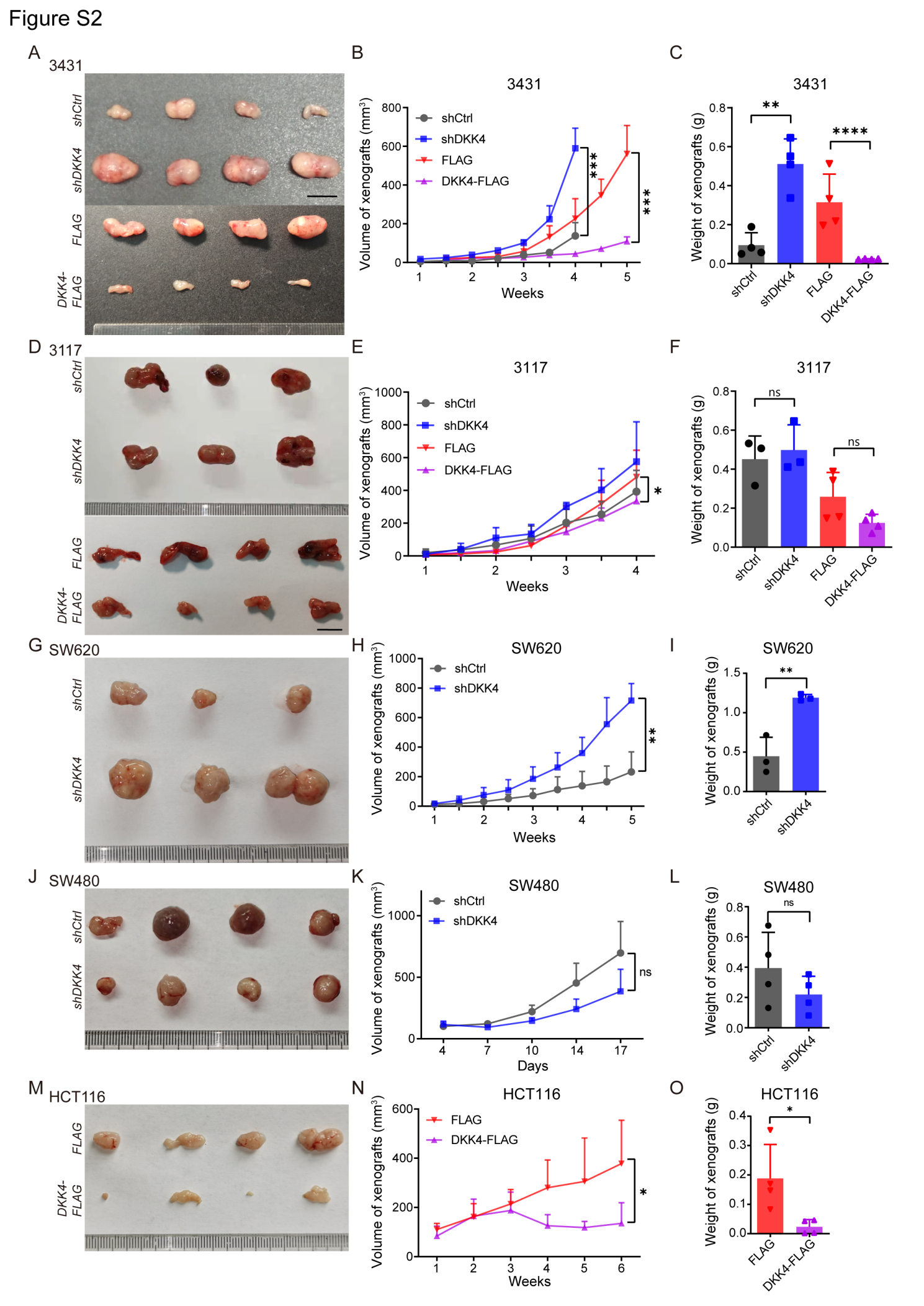


**Figure S2. DKK4 restricts the expansion of CRC xenografts in mice, related to Figure 2.**

(A-C) Tumour images (A), tumour volume (mm3) changing curves (B), and tumour weights (C) dissected from nude mice harbouring respective 3431-CCSCs subcutaneously (n = 4).

(D-F) Tumour images (D), tumour volume (mm3) changing curves (E), and tumour weights (F) dissected from nude mice harbouring respective 3117-CCSCs subcutaneously (n ≥ 3).

(G-I) Tumour images (G), tumour volume (mm3) changing curves (H), and tumour weights (I) dissected from nude mice harbouring respective SW620 cells subcutaneously (n = 3).

(J-L) Tumour images (J), tumour volume (mm3) changing curves (K), and tumour weights (L) dissected from nude mice harbouring respective SW480 cells subcutaneously (n = 4).

(M-O) Tumour images (M), tumour volume (mm3) changing curves (N), and tumour weights (O) dissected from nude mice harbouring respective HCT116 cells subcutaneously (n = 4).

Scale bars, 10 mm. Mean ± SD, *p < 0.05; **p < 0.01; ***p < 0.001; ****p < 0.0001 by Student’s unpaired t test.


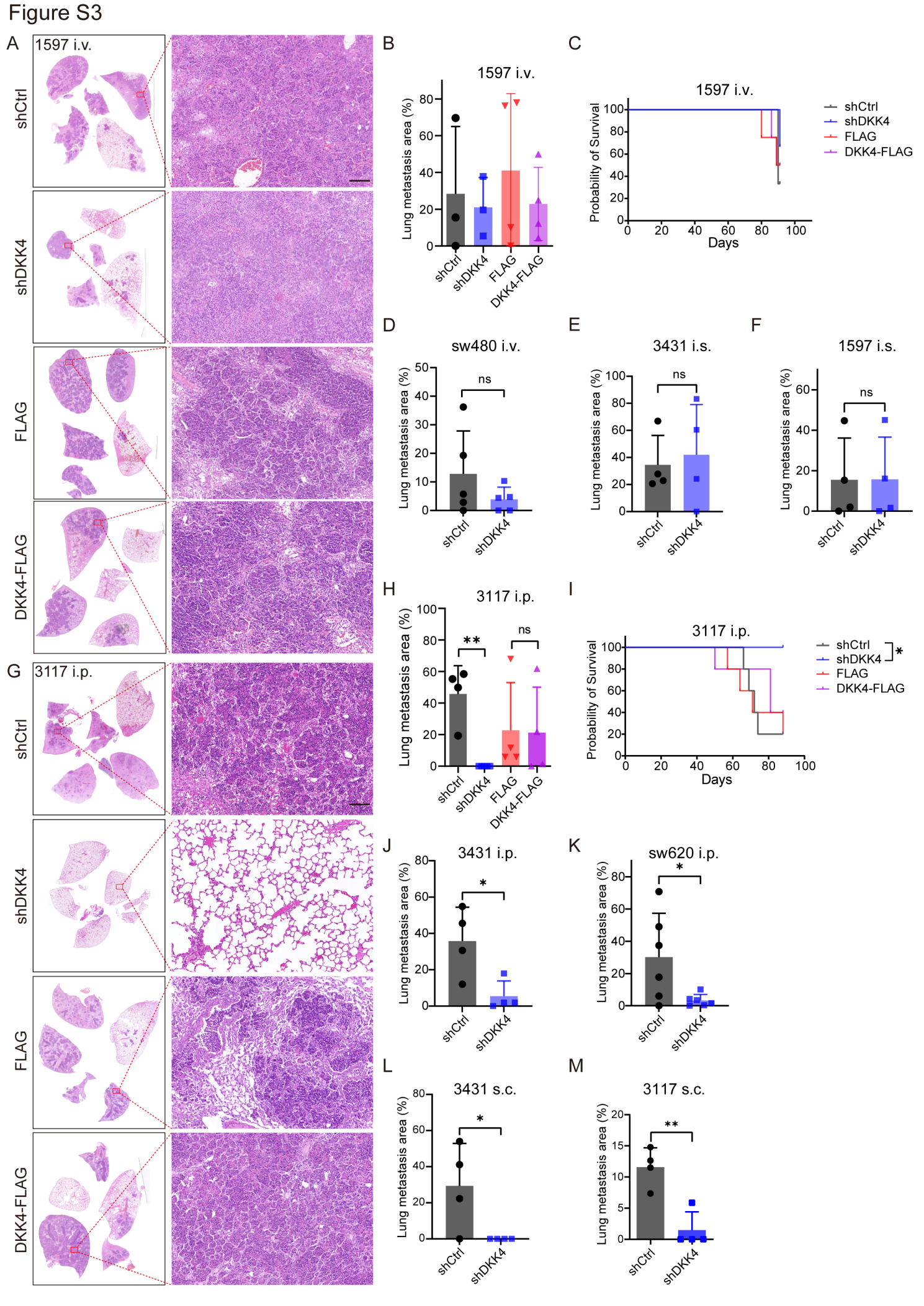


**Figure S3. A proper level of DKK4 promotes the metastasis of CRC xenografts in mice, related to Figure 2.**

(A-C) H&E images and quantification of lung metastasis (A and B) and survival curves (C) of nude mice harbouring respective 1597-CCSCs intravenously (i.v.) (shCtrl vs. shDKK4, n = 3; FLAG vs. DKK4-FLAG, n = 4).

(D) Quantification of lung metastasis in nude mice harbouring respective sw480 cells intravenously (i.v.) (n = 5).

(E and F) Quantification of lung metastasis in nude mice harbouring respective 3431-CCSCs (E) and 1597-CCSCs (F) through intrasplenic injection (i.s.) (n = 4).

(G-I) H&E images and quantification of lung metastasis (G and H) and survival curves (I) of nude mice harbouring respective 3117-CCSCs intraperitoneally (i.p.) (n = 4).

(J and K) Quantification of lung metastasis in nude mice harbouring respective 3431-CCSCs (J, n = 4) and SW620 cells (K, n = 6) intraperitoneally (i.p.).

(L and M) Quantification of lung metastasis in nude mice harbouring respective 3431-CCSCs (L, n = 4) and 3117-CCSCs (M, n = 4) subcutaneously (s.c.).

Scale bars, 100 μm. Mean ± SD, *p < 0.05; **p < 0.01; ***p < 0.001 by Student’s unpaired t test (B, D, E, F, J, K, and M), Mann-Whitney u test (H, L), and log-rank test (C and I).


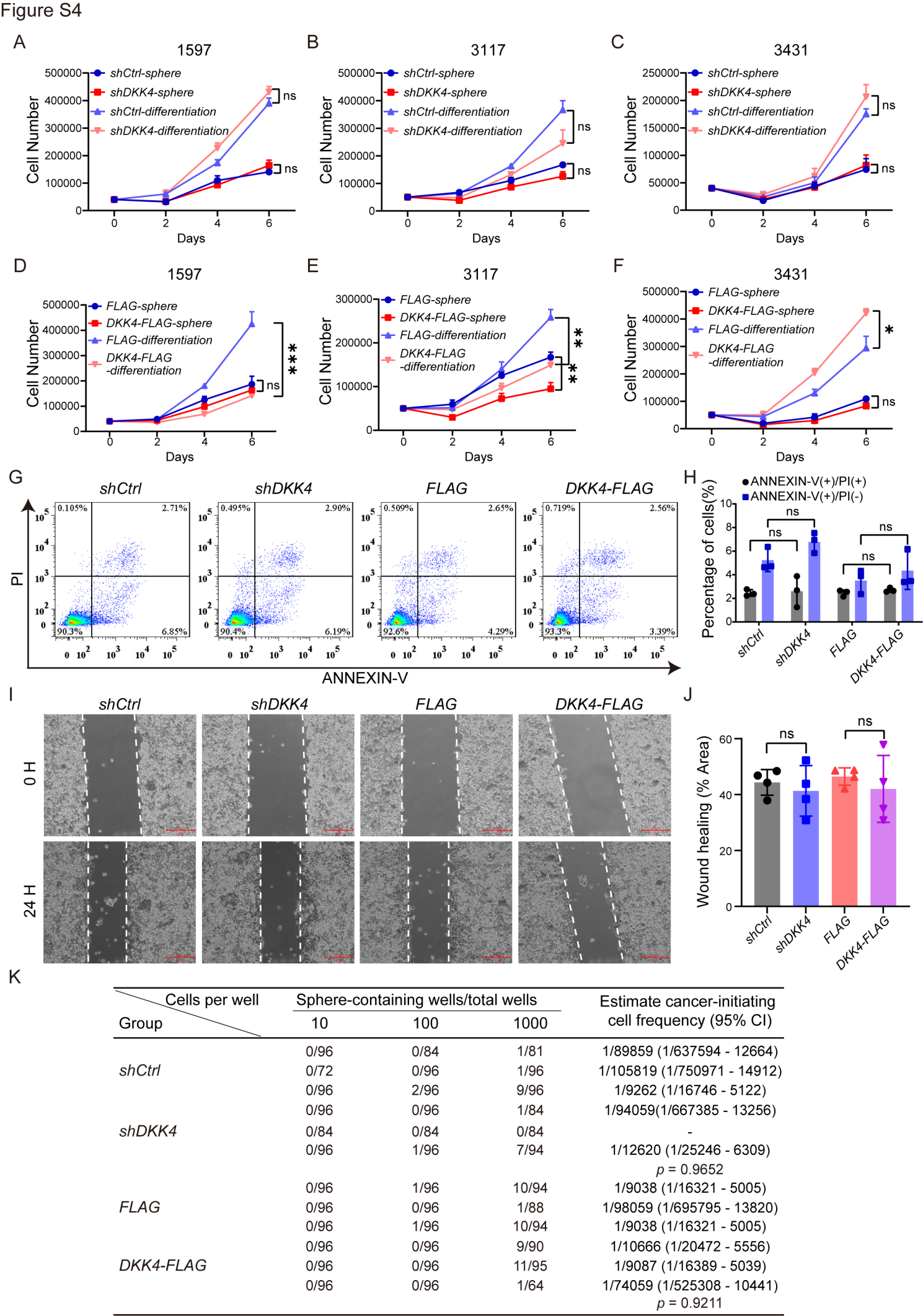


**Figure S4. DKK4 has no stable effect on CRC stem cell proliferation, apoptosis, migration ability and stemness of colorectal cancer cells.**

(A-C) Cell growth curves of shCtrl versus shDKK4 CCSCs cultured in sphere or differentiation culture medium. A,1597; B, 3117; and C, 3431 (n = 3).

(D-F) Cell growth curves of FLAG versus DKK4-FLAG CCSCs cultured in sphere or differentiation culture medium. D,1597; E, 3117; and F, 3431 (n = 3).

(G-H) Cell apoptosis assessment by ANNEXIN-V/PI double staining (G), and quantification (H) in each CCSCs (n = 3).

(I-J) Cell migration ability assessment by wound healing assay (I), and quantification (J) in each CCSCs (n = 4).

(K) Cancer-initiating cell frequency assay of xenografts harvested from each CCSCs-bearing mice.

Scale bars, 200 μm. At least three independent experiments were performed for each CCSCs. Mean ± SD, *p < 0.05; **p < 0.01; ***p < 0.001 by Student’s unpaired t test.


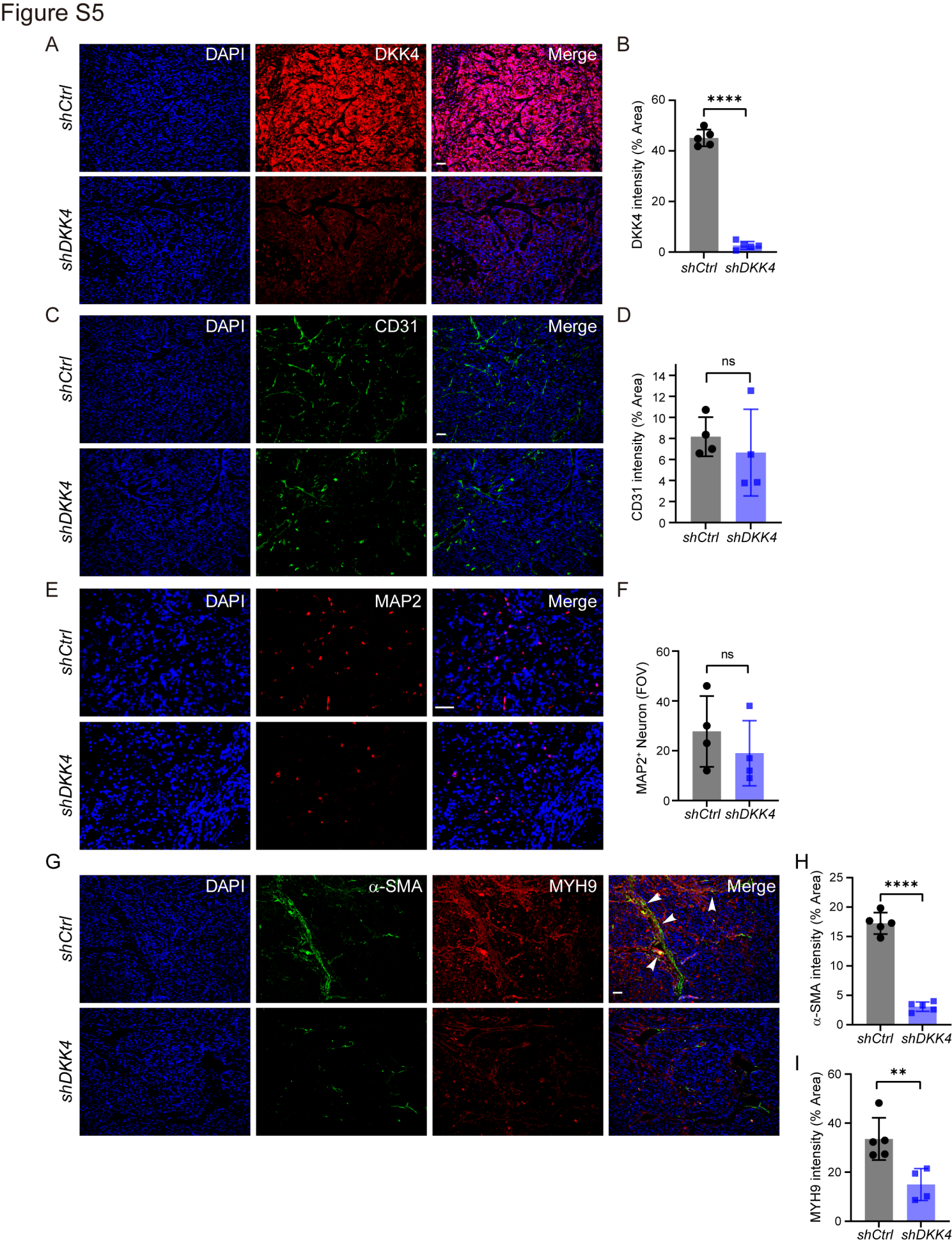


**Figure S5. DKK4 secreted from CRC cells transforms fibroblasts in stromal tissues of xenografts in mice, related to Figure 3.**

(A and B) Representative images (A) and quantification (B) of DKK4 (red) immunofluorescence staining in xenografts dissected from nude mice harbouring 3117-shCtrl or 3117-shDKK4 intraperitoneally (n = 5).

(C and D) Representative images (C) and quantification (D) of CD31 (green) immunofluorescence staining in xenografts (n = 4).

(E and F) Representative images (E) and quantification (F) of MAP2 (red) immunofluorescence staining in xenografts (n = 4).

(G-I) Representative images (G) and quantification of α-SMA (green, H), and MYH9 (red, I) immunofluorescence staining in xenografts. White arrows indicate myofibroblast (n = 5).

Scale bars, 50 μm. At least three independent experiments were performed. FOV, field of view. Mean ± SD, **p < 0.01; ****p < 0.0001 by Student’s unpaired t test.


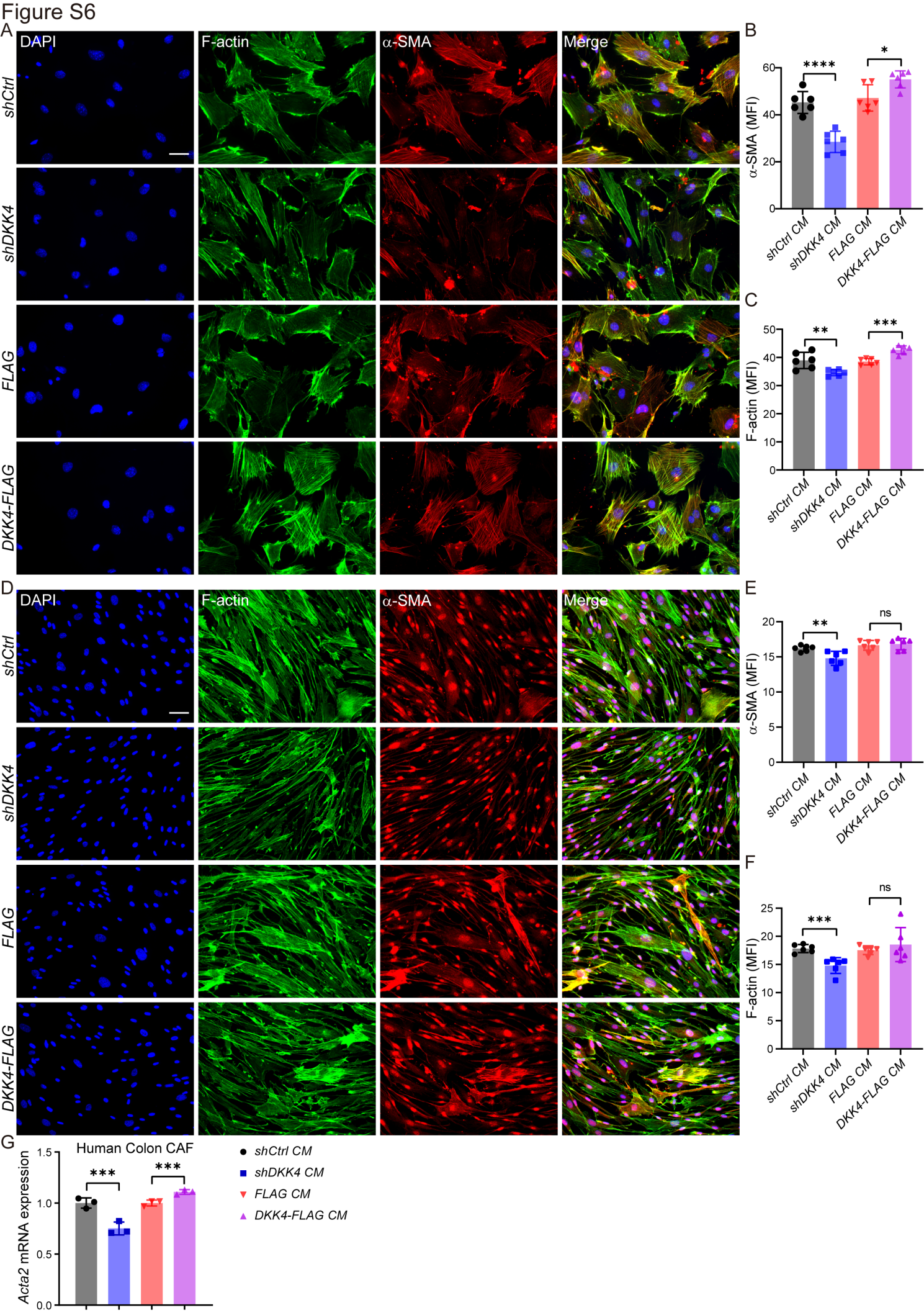


**Figure S6. DKK4 secreted from CRC cells transforms fibroblasts in vitro, related to Figure 4.**

(A-C) α-SMA (red) and F-actin (green) immunofluorescence staining (A), and quantification (B and C) in MEFs cultured with CM obtained from the respective 3431-CCSCs (containing 10% FBS) (n = 6).

(D-F) α-SMA (red) and F-actin (green) immunofluorescence staining (D), and quantification (E and F) in human colon CAFs cultured with CM obtained from the respective 1597-CCSCs (n = 6).

(G) Acta2 expression in human colon CAFs cultured with CM obtained from the respective 1597-CCSCs (n = 3).

Scale bars, 50 μm. MFI, mean fluorescence intensity. At least three independent experiments were performed. Mean ± SD, *p < 0.05; **p < 0.01; ***p < 0.001; ****p < 0.0001 by Student’s unpaired t test.


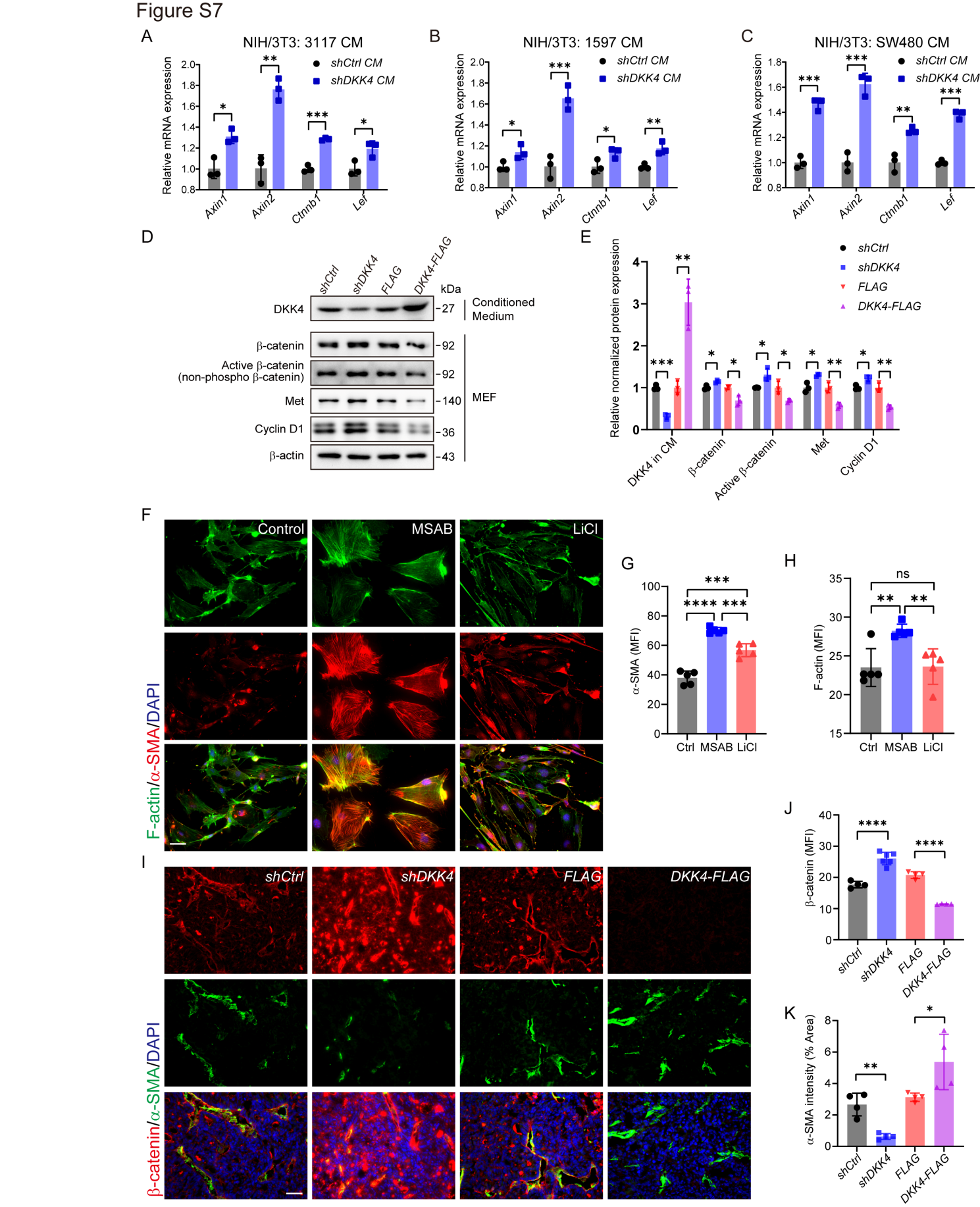


**Figure S7. β-catenin modulates fibroblast transformation signalling via DKK4, related to Figure 5.**

(A-C) Expression of Wnt/β-catenin signaling related genes in NIH3T3 cells cultured with shCtrl versus shDKK4 conditioned medium (CM) provided by 3117-CCSCs (A), 1597-CCSCs (B), and SW480 cells (C) (n = 3).

(D and E) Western blot images (D), and protein level quantification (E) of DKK4 in the CM obtained from the respective 1597-CCSCs, and Wnt/β-catenin signaling related genes (β-catenin, active β-catenin, Met and Cyclin D1) in MEFs cultured with each CM. Statistical analysis was carried out with three technical replicates using each protein sample.

(F-H) Representative images (F) and quantification of α-SMA (red, G) and F-actin (green, H) double staining in MEFs cultured with MSAB (2.5 μM), LiCl (20 mM) or equal volumes vehicle.

(I-K) Representative images (I) and quantification of β-catenin (red, J) and α-SMA (green, K) immunofluorescence staining in xenografts dissected from nude mice harbouring respective 1597-CCSCs.

Scale bars, 50 μm. MFI, mean fluorescence intensity. Three independent experiments were performed for each assessment. Mean ± SD, *p < 0.05; **p < 0.01; ***p < 0.001; ****p < 0.0001 by Student’s unpaired t test.


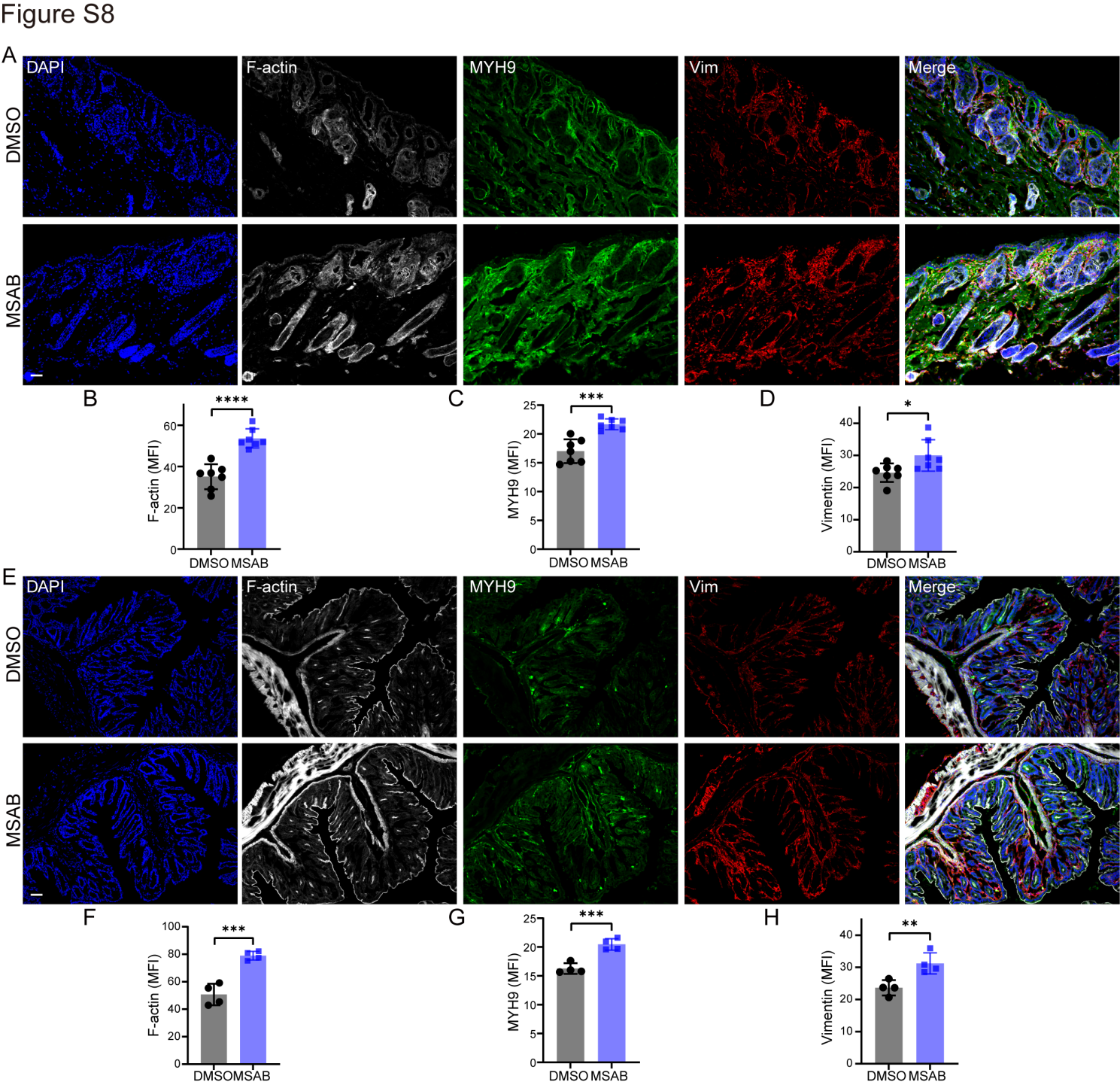


**Figure S8. β-catenin inhibitor modulates fibroblast transformation of skin and colorectal mucosa in mouse models, related to Figure 6.**

(A-D) Representative images (A) and quantification of F-actin (white, B), MYH9 (green, C) and Vimentin (red, D) triple staining in skin of nude mice administered with MSAB (20 mg/kg) or an equal volume of DMSO every two days (n = 6).

(E-H) Representative images (E) and quantification of F-actin (white, F), MYH9 (green, G) and Vimentin (red, H) triple staining in colorectal mucosa of nude mice related to (A-D) (n = 4).

Scale bars, 50 μm. MFI, mean fluorescence intensity. Three independent experiments were performed for each CCSCs. Mean ± SD, *p < 0.05; **p < 0.01; ***p < 0.001; ****p < 0.0001 by Student’s unpaired t test.

**
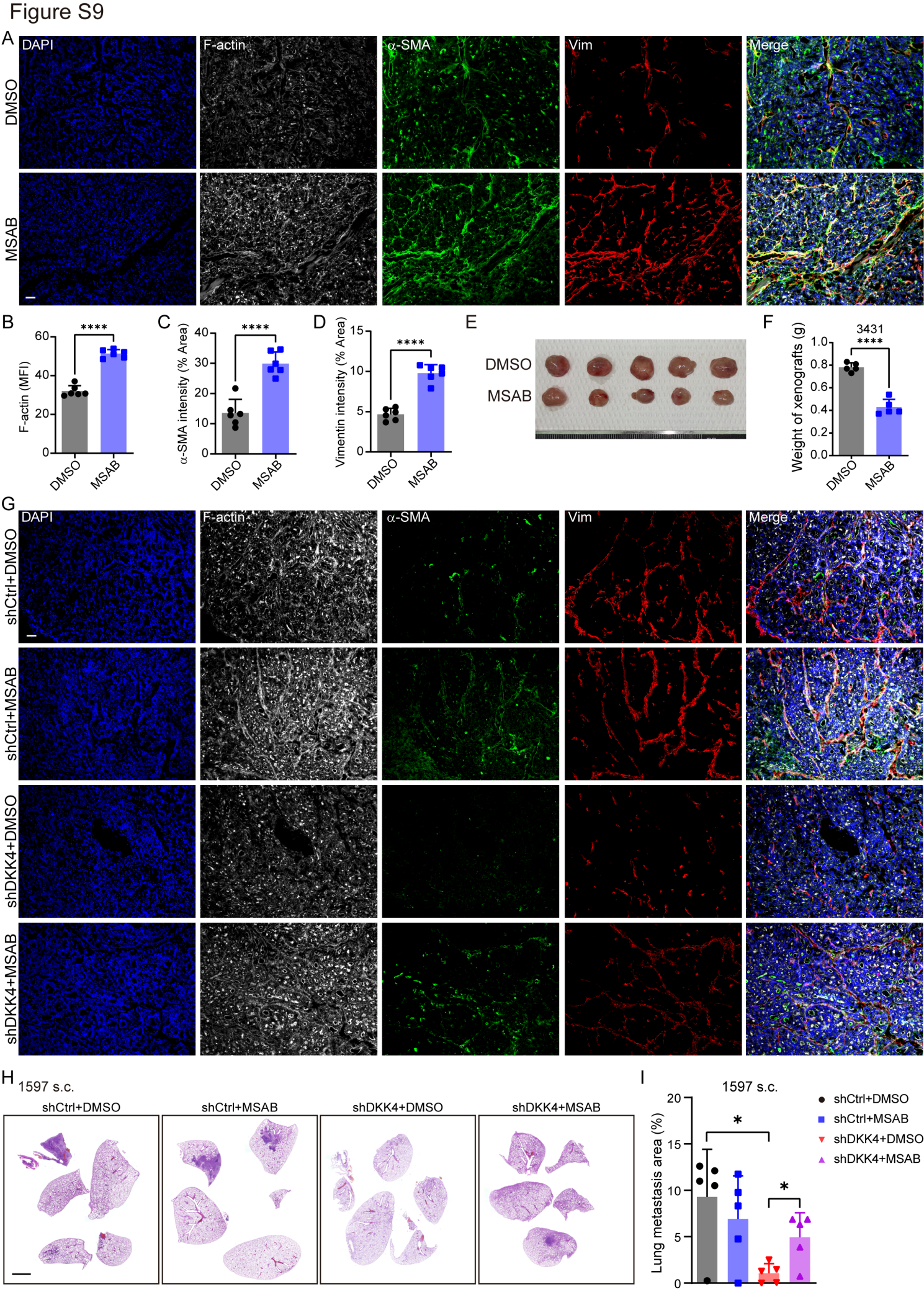
**

**Figure S9. β-catenin inhibitor modulates fibroblast transformation of xenografts in mouse models, related to Figure 6.**

(A-D) Representative images (A) and quantification of F-actin (white, B), α-SMA (green, C) and Vimentin (red, D) triple staining in xenografts dissected from nude mice harbouring 3431-CCSCs s.c., and MSAB (20 mg/kg) or an equal volume of DMSO was administered every two days (n = 6). Scale bar, 50 μm.

(E-F) Tumour images (E) and tumour weights (F) of harvested xenografts dissected from nude mice harbouring 3431-CCSCs s.c. and administered MSAB (20 mg/kg) or an equal volume of DMSO every two days (n = 5).

(G) Representative images of F-actin (white), α-SMA (green) and Vimentin (red) triple staining in xenografts dissected from nude mice harbouring 1597-CCSCs s.c., and MSAB (20 mg/kg) or an equal volume of DMSO was administered every two days (n = 5). Scale bar, 50 μm.

(H-I) H&E images (H) and quantification of lung metastasis (I) in nude mice harbouring the respective 1597-CCSCs s.c. and administered MSAB (20 mg/kg) or an equal volume of DMSO every two days (n = 5). Scale bar, 2000 μm.

MFI, mean fluorescence intensity. Mean ± SD, *p < 0.05; ****p < 0.0001 by Student’s unpaired t test.

**
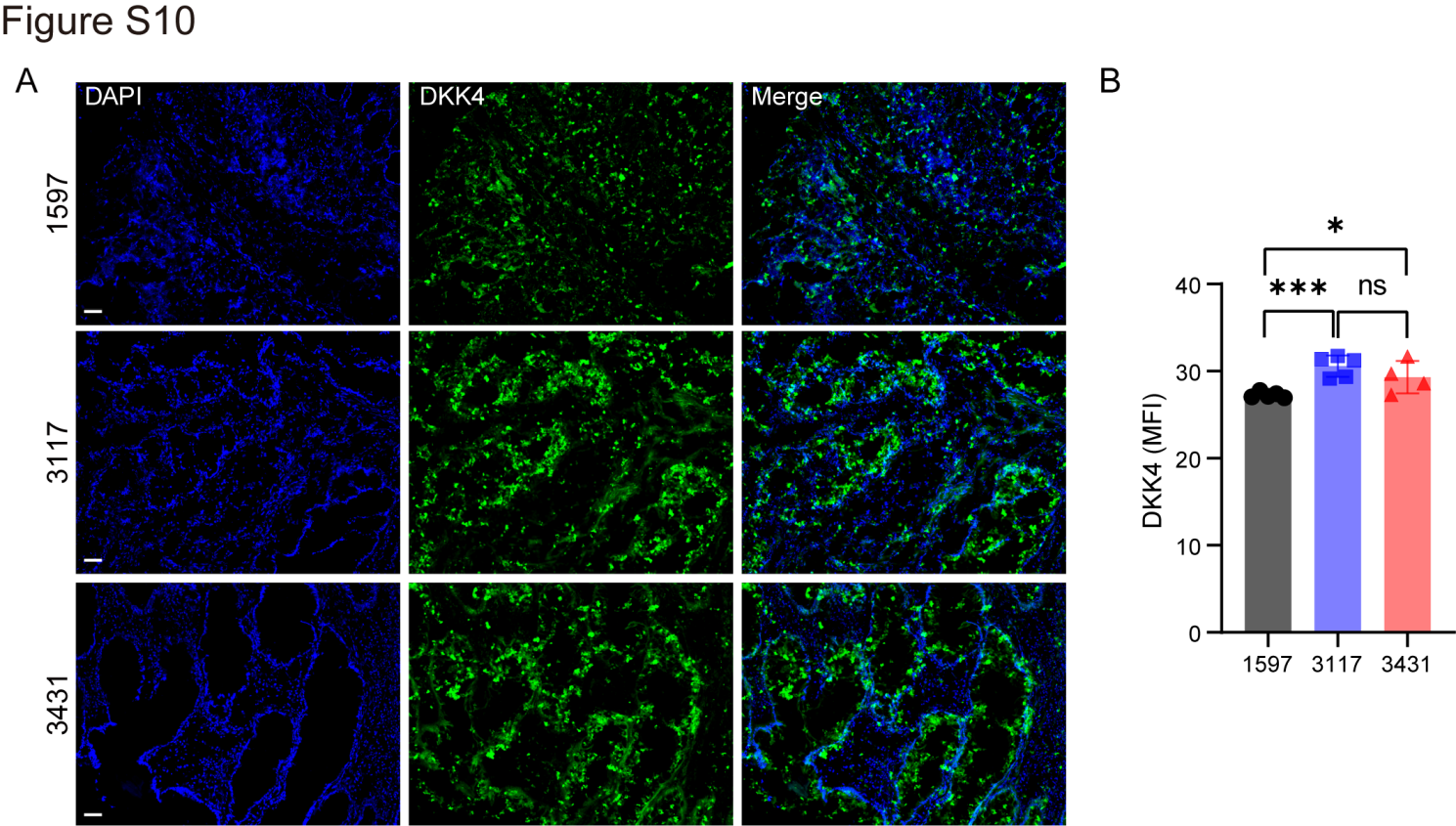
Figure S10. The expression of DKK4 protein in CRC tissue of patients.**

(A and B) Representative images (A) and quantification (B) of DKK4 (green) immunofluorescence staining in CRC tissue of patients (1597, 3117, and 3431). Scale bars, 50 μm.

MFI, mean fluorescence intensity. Mean ± SD, *p < 0.05; ***p < 0.001 by Student’s unpaired t test.

**Supplementary Tables**

Table S1. Targeting sequences for shRNA. DKK4-sh1 was valid and chose to be used for future experiments.

| Name | Sequence（5’—— 3’） |
| --- | --- |
| Scramble | CAACAAGATGAAGAGCACCAA |
| DKK4-sh1 | CACGGACTGCAATACCAGAAA |
| DKK4-sh2 | GTTTGTACTACGATGGAAGAT |
| DKK4-sh3 | GTGTGAACGATGTTTGTACTA |

Table S2. Primers for RT-PCR.

| Name | Sequence（5’—— 3’） |
| --- | --- |
| DKK4-F | CAACCCAAAAGGAAGCCAAG |
| DKK4-R | GCCACAGTCAAAAGTTCTCAG |
| GAPDH-F | ACATCGCTCAGACACCATG |
| GAPDH-R | TGTAGTTGAGGTCAATGAAGGG |
| ACTA2-F | AATGCAGAAGGAGATCACGG |
| ACTA2-R | TCCTGTTTGCTGATCCACATC |
| m/Acta2-F | GTGAAGAGGAAGACAGCACAG |
| m/Acta2-R | GCCCATTCCAACCATTACTCC |
| m/Axin1-F | AGTGATGCTGACACGCTATC |
| m/Axin1-R | GGTAAGTGCGAGGAATGTGAG |
| m/Axin2-F | TAGGTTCCGGCTATGTCTTTG |
| m/Axin2-R | TGTTTCTTACTCCCCATGCG |
| m/Col3a1-F | GAAGTCTCTGAAGCTGATGGG |
| m/Col3a1-R | TTGCCTTGCGTGTTTGATATTC |
| m/Col5a2-F | AGGAGAAGGAAACATCAGATTCAG |
| m/Col5a2-R | TTGCCAATATCCACAGGACC |
| m/Col6a1-F | CTGGTGAAGGAGAACTATGCAG |
| m/Col6a1-R | GTCTAGCAGGATGGTGATGTC |
| m/Col10a1-F | CATCTCCCAGCACCAGAATC |
| m/Col10a1-R | CCCATGAACCAGGGTCAAGAA |
| m/Ctnnb1-F | GCTATTCCACGACTAGTTCAGC |
| m/Ctnnb1-R | AGCTCCAGTACACCCTTCTAC |
| m/Gapdh-F | GTGAACCACGAGAAATATGACAAC |
| m/Gapdh-R | AGTGATGGCATGGACTGTG |
| m/Lef1-F | AGCCTGTTTATCCCATCACG |
| m/Lef1-R | TGTTACAATAGCTGGATGAGGG |

m: mouse gene.
